# Necessity of p53-binding to the *CDH1* locus for its expression defines two epithelial cell types differing in their integrity

**DOI:** 10.1038/s41598-018-20043-7

**Published:** 2018-01-25

**Authors:** Tsukasa Oikawa, Yutaro Otsuka, Yasuhito Onodera, Mei Horikawa, Haruka Handa, Shigeru Hashimoto, Yutaka Suzuki, Hisataka Sabe

**Affiliations:** 1 Department of Molecular Biology, Graduate School of Medicine, Hokkaido University, North 15, West 7, Kita-ku, Sapporo, Hokkaido, 060-8638 Japan; 2 Laboratory of Functional Genomics, Department of Medical Genome Sciences, Graduate School of Frontier Science, The University of Tokyo, 5-1-5 Kashiwanoha, Kashiwa, Chiba, 277-8561 Japan

## Abstract

*TP53* mutation (*i.e*., loss of normal-p53) may evoke epithelial-mesenchymal transition (EMT), which was previously attributed to loss of certain miRNAs. However, not all epithelial cells undergo EMT upon *TP53* mutation, and the p53-miRNA axis may not fully explain p53 function in epithelial integrity. We here show two modes of epithelial integrity: one involves p53-binding to a nucleotide region and the other does not. In the former, p53 binds to the *CDH1* (encoding E-cadherin) locus to antagonize EZH2-mediated H3K27 trimethylation (H3K27me3) to maintain high levels of acetylation of H3K27 (H3K27ac). In the latter, the same locus is not highly acetylated at H3K27, and does not allow p53-binding, nor needs to antagonize EZH2. We moreover demonstrated that although the *CDH1* locus in the p53-independent cells, but not in fibroblasts, becomes high-H3K27ac by butyrate and allows p53-biniding, their *CDH1* expression does not become dependent on p53. Our results identified novel modes of the epithelial integrity, in which the same epithelial-specific gene locus exhibits different requirement for p53 with different histone modifications among different epithelial cells to warrant its expression.

## Introduction

p53, the *TP53* gene product, is a pleiotropic protein with functions that appear to culminate in maintaining genome integrity, such as by acting as a transcriptional cofactor^1^, regulating cellular metabolic reprograming to maintain antioxidative statuses^2–6^, and sometimes by eliminating severely damaged cells^7^. On the other hand, p53 also appears to play a role in maintaining epithelial integrity. It has been shown that *TP53* mutation, or loss of normal-p53 often evokes mesenchymal phenotypes of breast cancer cells and lung cancer cells, to be often coupled with the acquisition of cancer stem cell-like cell properties^8,9^. As for a molecular mechanism therein involved, it was shown previously that normal-p53 has a potential to induce certain microRNAs (miRNAs) that target mRNAs encoding transcription factors (TFs) driving epithelial-mesenchymal transition (EMT), such as *ZEB1*, *SNAI1*, and *BMI1*^10,11^. However, it is well documented that not all epithelial cells undergo EMT upon loss of normal-p53^12,13^. Moreover, given the cell context-dependent expression of those EMT-TFs^14^ as well as a significant enrichment of the putative p53-binding motifs in the regulatory regions of epithelial-specific genes^15–17^, p53 seems to have further unidentified functions specific to epithelial cells.

We here found that p53 binds to nucleotides of the *CDH1* locus (encoding E-cadherin) in certain epithelial cells, in which p53-binding is necessary to maintain *CDH1* expression and epithelial integrity (in this paper we call them EMT-prone cells), whereas p53 does not bind to the same nucleotide region of the *CDH1* locus in other epithelial cells that do not require p53 to maintain *CDH1* expression (*i.e*., EMT-resistant cells). Histone modifications at the *CDH1* locus are significantly different between these two types of cells. Together with detailed mechanisms, we identified a novel mechanism by which p53 acts to maintain *CDH1* expression and the epithelial integrity. Our results suggested that in addition to the p53-miRNA axis, at least two other mechanisms exist with regard to maintaining *CDH1* expression in epithelial cells, which may be important to block unnecessary onset of EMT.

## Results

### Requirement of p53 for E-cadherin expression without suppressing ZEB1

**Figure 1:**
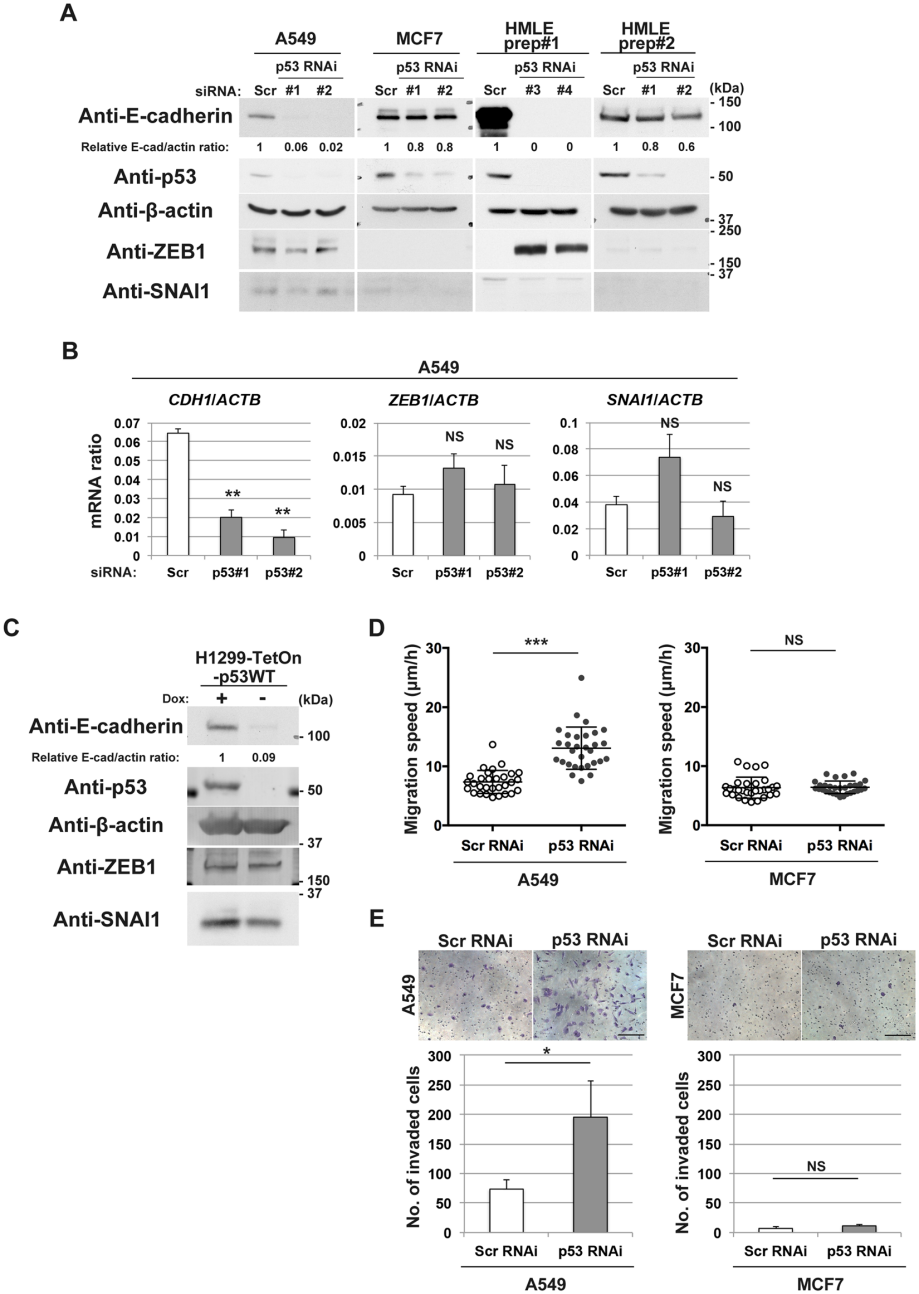
p53 maintains E-cadherin expression without ZEB1 or SNAI1 in A549 cells and H1299 cells. (**A**) A549 cells, MCF7 cells, or HMLE cells transduced with scramble (Scr) or p53 (#1 or #2) siRNA, or p53 shRNA (#3 or #4) were subjected to immunoblot analysis with the indicated antibodies. E-cadherin and β-actin bands (E-cad and actin, respectively) were quantified using Image J software, and normalized E-cad/actin ratios are indicated. (**B**) A549 cells transfected with scramble (Scr) or p53 (#1 or #2) siRNA were also subjected to quantitative RT-PCR analysis of *CDH1*, *ZEB1*, or *SNAI1* mRNA (normalized to *ACTB* mRNA). Data are means ± SD of 3 independent experiments. **P < 0.001; NS, not significant versus Scr (Student *t*-test). (**C**) H1299-TetOn-p53WT cells cultured in the presence (+) or absence (−) of doxycycline (Dox) (0.5 µg/ml) for 72 h were subjected to immunoblot analysis with the indicated antibodies. E-cadherin and β-actin bands (E-cad and actin, respectively) were quantified using Image J software, and normalized E-cad/actin ratios are indicated. (**D**) A549 or MCF7 cells transfected with scramble (Scr) or p53 siRNA were subjected to live-cell imaging analysis. Velocity of migration obtained by the total track distance of each cell divided by time is indicated. Bars represent means ± SD. ***P < 0.0001; NS, not significant versus Scr (Student *t*-test). (**E**) A549 or MCF7 cells transfected with scramble (Scr) or p53 siRNA were replated in Matrigel chambers and assayed for invasive activity. Cells that had invaded through the Matrigel at 24 h after plating were stained with crystal violet (top), and those in three different sampling areas were counted. Bar, 200 µm. Quantitative data are means ± SD from three independent experiments. *P < 0.05; NS, not significant versus Scr (Student *t*-test). Western blots were cropped for clarity; uncropped images are shown in Supplementary Figure S5.

Normal-p53 is crucial for E-cadherin expression in MCF12A mammary epithelial cells, in which normal-p53 acts to suppress expression of *ZEB1* via certain miRNA, in order to maintain E-cadherin expression^10,11^. Likewise, we found that p53 also appears to be essential for E-cadherin expression in A549 lung cancer cells, in which siRNA-mediated silencing of *TP53* abolished the E-cadherin expression (Fig. 1A). However, *ZEB1*, both its mRNA and protein, was already expressed in A549 cells at a low level, and not notably increased upon *TP53* silencing (Fig. 1A,B). *SNAI1* mRNA and protein levels were also not significantly increased by *TP53* silencing (Fig. 1A,B). We also found that introduction of normal-p53 (p53WT) into p53-deficient H1299 lung cancer cells restored their E-cadherin expression without suppressing ZEB1 or SNAI1 (Fig. 1C). These results implied that suppression of EMT-TFs, such as ZEB1, by p53 might not be the entire mechanism by which normal-p53 maintains E-cadherin expression in epithelial cells.

### p53-independent maintenance of E-cadherin expression

We then found that silencing of *TP53* does not notably affect E-cadherin expression in MCF7 breast cancer cells (Fig. 1A). These cells did not express ZEB1 or SNAI1 at detectable levels (Fig. 1A). HMLE cells are immortalized populations of primary human mammary epithelial cells, by use of SV40 large T antigen and human telomerase reverse transcriptase^18^. It has been reported that HMLE cells may have intrinsic heterogeneity with regard to their cell phenotypes^9^. We found that different preparations of HMLE cells exhibit different requirement for p53 in their E-cadherin expression: the preparation #1 of HMLE cells (prep#1) need p53 for E-cadherin expression, whereas the preparation #2 cells (prep#2) do not (Fig. 1A). The prep#2 cells did not express ZEB1 or SNAI1 at detectable levels as in the case with MCF7 cells, whereas ZEB1 became clearly induced upon loss of normal-p53 in the prep#1 cells as in the case with MCF12A cells^10^. These results indicated that some epithelial cells do not require p53 for their E-cadherin expression.

Loss of E-cadherin expression in epithelial cells is a hallmark of their onset of the EMT program, which promotes cell motile activities such as migration and invasion^19^. We found that the silencing of *TP53* did not promote migration and invasion of MCF7 cells, whereas this silencing promoted migration and invasion of A549 cells (Fig. 1D,E). Together with above results, our results indicated that some epithelial cells, like MCF7 cells, do not need intact *TP53* to prohibit the onset of the EMT program, while some others, like A549 cells, indeed need this genome guardian to block EMT.

### p53 opposes trimethylation of H3K27 to maintain its acetylation at the *CDH1* locus in EMT-prone cells

**Figure 2:**
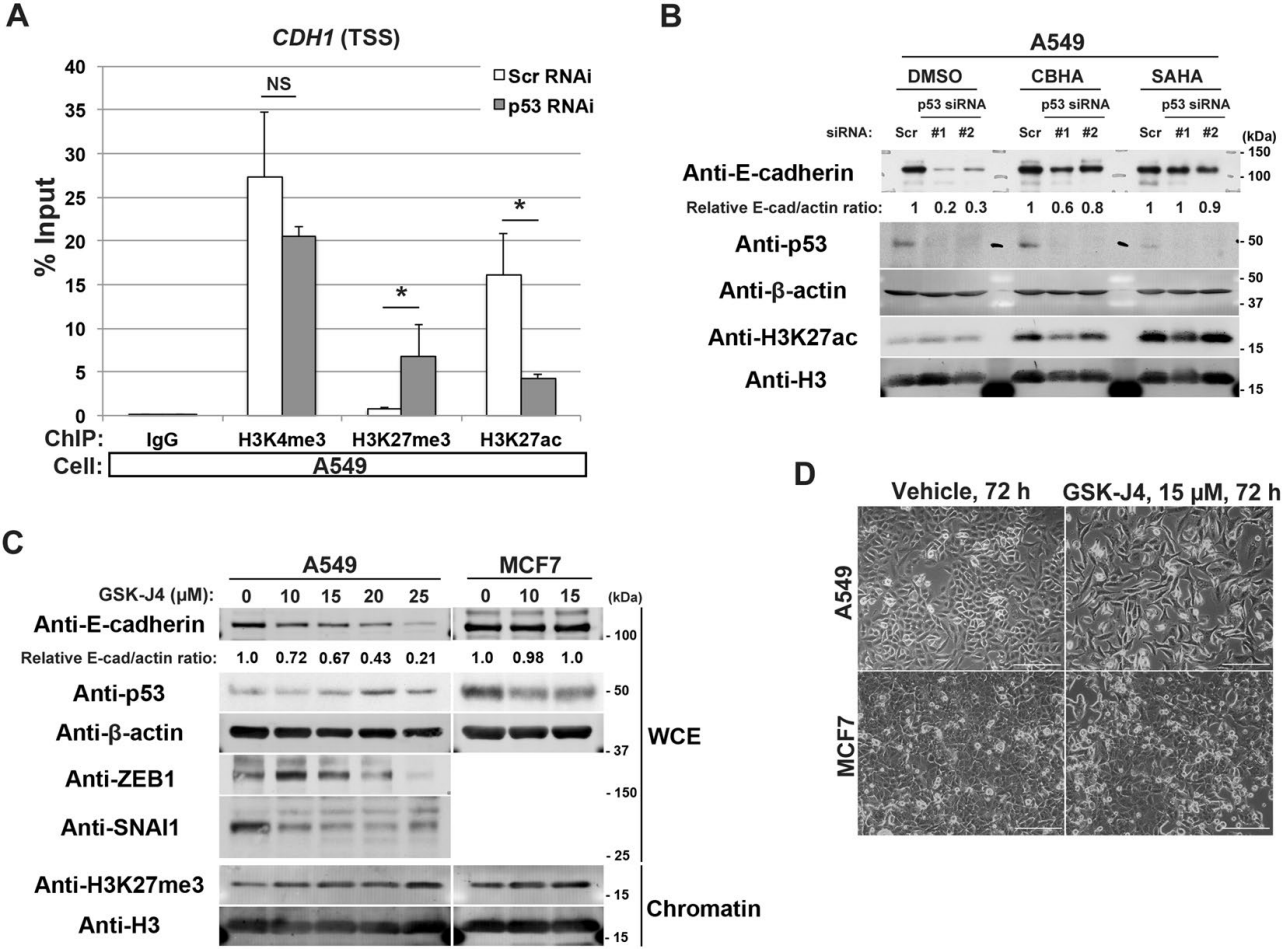
Loss of p53 leads to epigenetic alterations around the *CDH1* TSS in A549 cells. (**A**) A549 cells transfected either with the scramble (Scr RNAi) or p53 (p53 RNAi) siRNA were subjected to ChIP using antibodies against the indicated modified histones, followed by quantitative RT-PCR analysis with the primers located near the TSS of the *CDH1* locus. Data are means ± SD of 3 independent experiments. *P < 0.05; NS, not significant vs Scr RNAi (Student *t*-test). (**B**) A549 cells transfected with scramble (Scr) or p53 (#1 or #2) siRNA were then treated with 2.5 µM of CBHA or 3.0 µM of SAHA for 30 h. Whole cell extracts were then subjected to immunoblot analysis with the indicated antibodies. E-cadherin and β-actin bands (E-cad and actin, respectively) were quantified using Image J software, and normalized E-cad/actin ratios are indicated. (**C**) A549 cells and MCF7 cells were treated with the indicated concentrations of GSK-J4 for 72 h. Whole cell extracts (WCE) or 1.5 µg of chromatin fractions (Chromatin) were subjected to immunoblot analysis with the indicated antibodies. Quantitative western blots of E-cadherin and β-actin (E-cad and actin, respectively) were performed with the infrared fluorescence imaging system on an Odyssey imager, and normalized E-cad/actin ratios are indicated. (**D**) Phase contrast images of A549 cells or MCF7 cells treated with 15 µM of GSK-J4 for 72 h. Bars, 200 µm. Western blots were cropped for clarity; uncropped images are shown in Supplementary Figure S5.

We then analysed whether p53 is involved in histone modifications of the *CDH1* locus in A549 cells. We analysed epigenetic statuses around the transcription start site (TSS) of the *CDH1* locus by a chromatin immunoprecipitation-quantitative PCR (ChIP-qPCR) method. Silencing of *TP53* in these cells caused a significant reduction in H3K27ac levels around the TSS region of *CDH1*, to be accompanied with an increment in H3K27me3 (Fig. 2A). H3K4me3 of this region was not changed significantly (Fig. 2A). M-carboxycinnamic acid bis-hydroxamide (CBHA) and suberoylanilide hydroxamic acid (SAHA) are inhibitors of histone deacetylases (HDACs)^20,21^. These inhibitors sustained E-cadherin expression in A549 cells even in the absence of p53 (Fig. 2B). These results collectively suggested that p53 is necessary to maintain high levels of acetylation of H3K27, perhaps by antagonizing the trimethylation, at the *CDH1* locus in A549 cells to warrant its expression.

p53 is known to interact with the histone lysine demethylase JMJD3/KDM6B^22^. We then sought to examine whether this demethylase is crucial for blocking H3K27me3 deposition at the *CDH1* locus in A549 cells, in cooperation with p53. We however found that p53 expression was largely reduced upon the silencing of *JMJD3/KDM6B* in A549 cells (Supplementary Figure S1), as has been reported previously with other cells^23,24^. Alternatively, we used GSK-J4, an inhibitor of H3K27me3-specific demethylases^25^. Treatment of A549 cells by this inhibitor drastically reduced E-cadherin expression in a dose-dependent manner without increasing the expression of ZEB1 or SNAI1 (Fig. 2C). These GSK-J4-treated cells showed a fusiform morphology, to be indicative of their conversion into a mesenchymal morphology (Fig. 2D). On the other hand, E-cadherin expression and cell morphology of MCF7 cells were not notably affected by this inhibitor (Fig. 2C,D). Therefore, the *CDH1* locus in A549 cells seems to be a constant target of H3K27 trimethylation, which some H3K27me3-specific demethylase(s) needs to antagonize. JMJD3/KDM6B, which is known to interact with p53, may or may not be involved in this demethylation. In contrast, the *CDH1* locus in MCF7 cells did not seem to be such a target of H3K27 trimethylation, nor require demethylase activities to maintain its expression.

### p53 antagonizes EZH2 to block H3K27me3 deposition in EMT-prone cells

**Figure 3:**
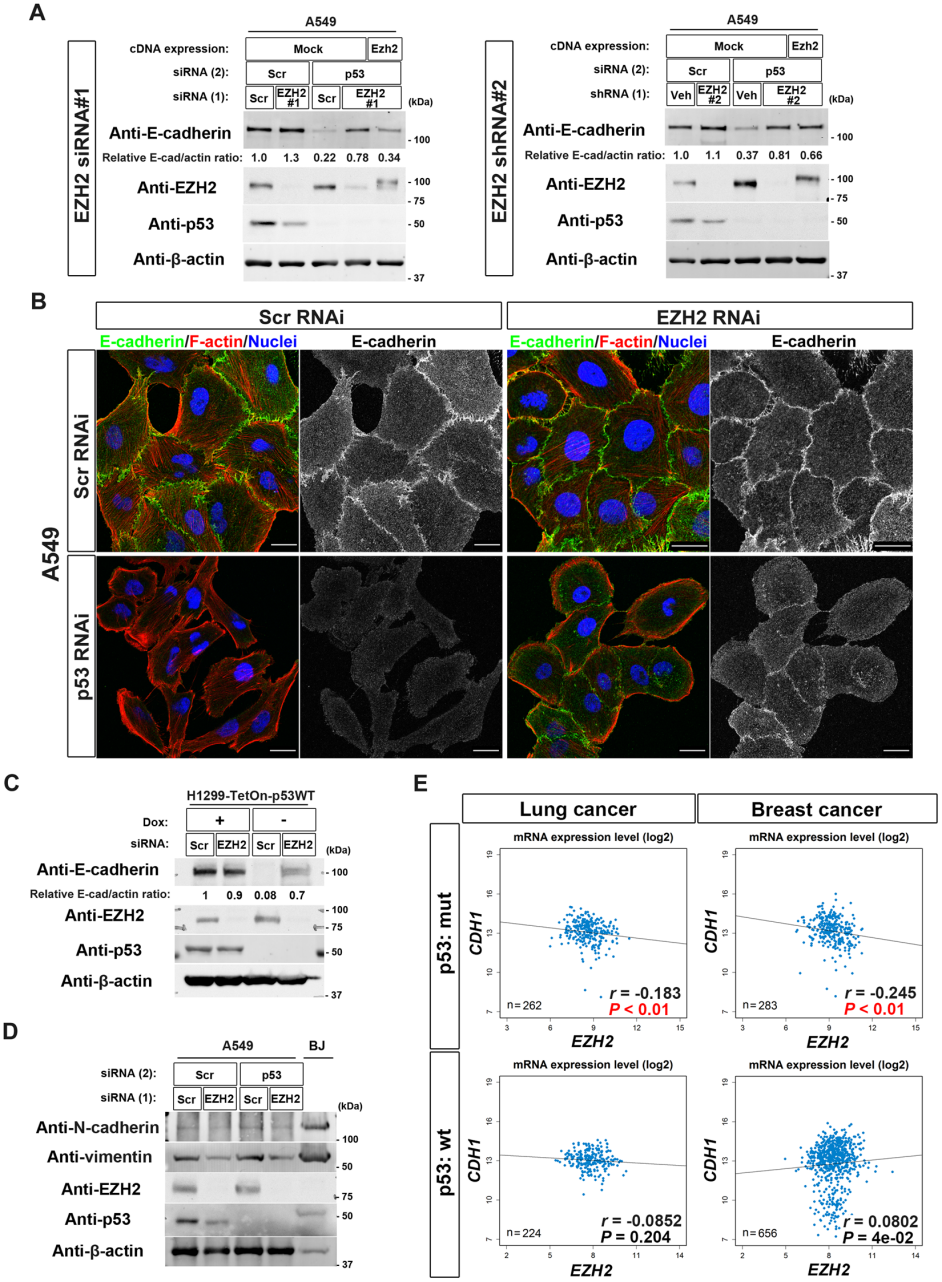
EZH2 mediates loss-of-p53-induced E-cadherin suppression in EMT-prone cells. (**A**) A549 cells reverse-transfected either with scramble (Scr) or EZH2 (EZH2 #1) siRNA (left) or transduced with lentiviruses expressing either vehicle (Veh) or shEZH2 (EZH2 #2) (right) were then transfected with scramble (Scr) or p53 siRNA (p53). The cells were then replated and reverse-transfected with mock or FLAG-HA-tagged mouse Ezh2 cDNA (Ezh2). After culturing for 72 h, the cells were subjected to immunoblot analysis with the indicated antibodies. Quantitative western blots of E-cadherin and β-actin were performed with the infrared fluorescence imaging system on an Odyssey imager. The normalized E-cadherin/β-actin (E-cad/actin) ratios are indicated. (**B**) A549 cells treated as in (**A**) were cultured for 72 h before fixation. The cells were stained with Texas Red–phalloidin (red) to detect F-actin, with antibodies to E-cadherin (green), and with DAPI (blue) to visualize nuclei. Bars, 20 µm. (**C**) H1299-TetOn-p53WT cells transfected with scramble (Scr) or EZH2 siRNA were cultured in the presence (+) or absence (−) of doxycycline (Dox) (0.5 µg/ml) for 48 h. The cells were then subjected to immunoblot analysis with the indicated antibodies. E-cadherin and β-actin bands (E-cad and actin, respectively) were quantified using Image J software, and normalized E-cad/actin ratios are indicated. (**D**) A549 cells treated as in (**A**) and BJ fibroblast cells were subjected to immunoblot analysis with the indicated antibodies. (**E**) Dot plots of TCGA RNA expression profiles of *CDH1* and *EZH2* in lung cancer or breast cancer with wild-type (wt) p53 or mutant (mut) p53 are shown. The Spearman rank correlation test was performed to analyse the statistical significance. A *P*-value of less than 0.05 for the inverse correlation is shown in red. Western blots were cropped for clarity; uncropped images are shown in Supplementary Figure S5.

We then sought to identify H3K27-specific methyltransferase(s) that may constantly target the *CDH1* locus, and from which p53 needs to protect this locus in A549 cells. PRC2 is a major H3K27-specific methyltransferase complex, in which EZH2 is the catalytic subunit^26^. We found that A549 cells maintain their E-cadherin expression even in the absence of p53, if their *EZH2* is silenced (EZH2 siRNA#1 and shRNA#2; Fig. 3A). Consistently, the silencing of *EZH2* cancelled the appearance of the fusiform morphology induced in the *TP53*-silenced A549 cells (Fig. 3B). Therefore, PRC2 appeared to be the H3K27-specific methyltransferase which p53 antagonizes to block H3K27me3 deposition at the *CDH1* locus in A549 cells. Another EMT-prone cells, H1299 cells also restored their E-cadherin expression when their *EZH2* is silenced (Fig. 3C). On the other hand, the silencing of *TP53* did not affect the expression of mesenchymal proteins such as N-cadherin and vimentin in A549 cells, although the expression of vimentin was attenuated upon *EZH2* silencing (Fig. 3D). ChIP-sequencing (ChIP-Seq) analysis also revealed that histone modifications at the mesenchymal-specific gene loci, such as *CDH2* (encoding N-cadherin) and *VIM* (encoding vimentin), as well as genes encoding EMT-TFs, such as *ZEB1*, *ZEB2*, *SNAI1*, *SNAI2*, *TWIST1*, and *TWIST2* were not notably altered upon *TP53* silencing (Supplementary Figure S2A). These data suggest that the p53-PRC2 antagonism primarily regulates the epithelial gene expression such as *CDH1*, but not the mesenchymal gene expression, in EMT-prone cells. We also noticed by analyzing The Cancer Genome Atlas (TCGA) RNA-Seq datasets of lung cancer and breast cancer that although *CDH1* mRNA levels are inversely correlated with *EZH2* mRNA levels in cells bearing *TP53* mutation, such a correlation is not observed in cells bearing intact *TP53* (Fig. 3E). This information is consistent with a notion that normal-p53 may protect the *CDH1* locus from the activity of EZH2, to warrant its expression at least in some population of lung cancer cells and breast cancer cells.

On the other hand, it was reported previously that the expression of EZH2 is augmented in the absence of normal-p53^27–29^. We however did not observe a notable change of EZH2 levels upon *TP53* silencing in A549 cells (Fig. 3A) or expression of normal-p53 in H1299 cells (Fig. 3C). TCGA RNA-Seq datasets also indicated that *EZH2* mRNA levels are not drastically changed, while slightly augmented, in cells bearing *TP53* mutation as compared to those in cells bearing intact *TP53* (Supplementary Figure S2B).

### p53 binds to the *CDH1* locus in EMT-prone cells but not in EMT-resistant cells

**Figure 4:**
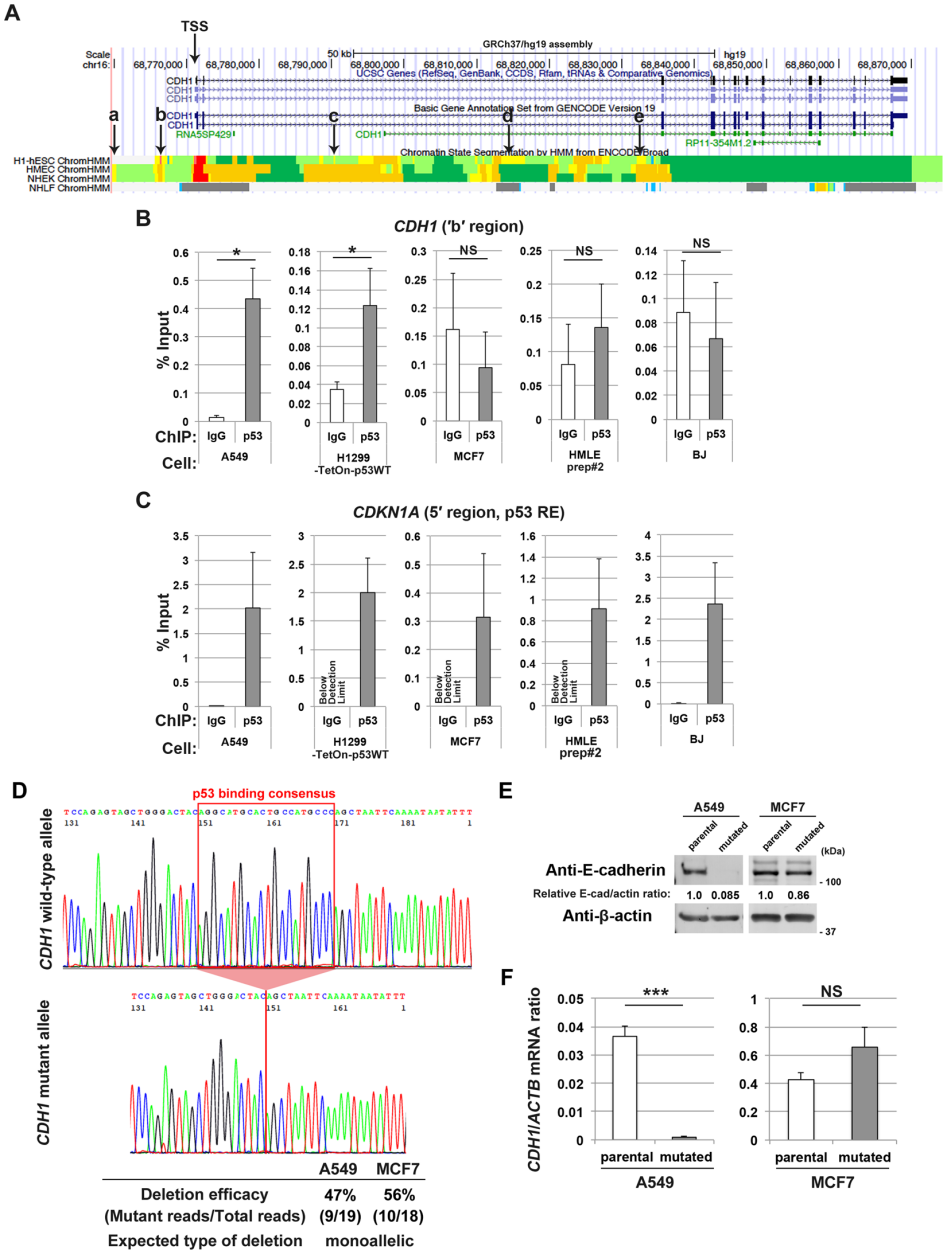
p53 binds to the *CDH1* locus to maintain its expression in EMT-prone cells. (**A**) UCSC Genome Browser view of the ENCODE data at the *CDH1* locus. The TSS and the p53 consensus binding motifs (a–e) across the *CDH1* locus are indicated. (**B,C**) ChIP was performed using A549 cells, H1299-TetOn-p53WT cells cultured in the presence of doxycycline (0.5 µg/ml) for 48 h, MCF7 cells, HMLE prep#2 cells, or BJ cells with a normal rabbit IgG or anti-p53 antibody, followed by quantitative RT-PCR analysis with primers located at the ′b′ region of *CDH1* (**B**) or at the p53 response element (RE) of *CDKN1A* (**C**). Data are means ± SD of 3 independent experiments. *P < 0.05, NS, not significant versus corresponding IgG control (Student *t*-test). (**D**) Example chromatogram showing successful deletion of the p53-binding consensus. Efficiency of the targeted deletion in A549 cells or MCF7 cells determined by sequencing is indicated. (**E**) A549 cells or MCF7 cells with (mutated) or without (parental) deletion of the p53-binding motif were subjected to immunoblot analysis with the indicated antibodies. Quantitative western blots of E-cadherin and β-actin (E-cad and actin, respectively) were performed with the infrared fluorescence imaging system on an Odyssey imager, and normalized E-cad/actin ratios are indicated. F, A549 cells or MCF7 cells as in (**E**) were subjected to quantitative RT-PCR analysis of *CDH1* mRNA (normalized to *ACTB* mRNA). Data are means ± SD of 3 independent experiments. ***P < 0.0001; NS, not significant (Student *t*-test). Western blots were cropped for clarity; uncropped images are shown in Supplementary Figure S5.

We then sought to understand the molecular basis as to why p53 is required for E-cadherin expression in some epithelial cells, but not in other cells. Using the “p53scan” algorithm^30^, we found several p53 consensus binding motifs across the regulatory nucleotide regions of the *CDH1* locus (Fig. 4A and Supplementary Figure S3A, a–e), as has been suggested by the ENCODE project^15,16^. We then found that p53 clearly binds to the ‘b’ region (GRCh37 chr16: 68,767,163–68,767,182) in A549 cells and H1299 cells that express normal-p53 (Fig. 4A,B and Supplementary Figure S3A), whereas p53-binding to this site was undetectable above the background in MCF7 cells, HMLE prep#2 cells, and BJ fibroblast cells (Fig. 4B). As a control, p53-binding to the *CDKN1A* promoter^31^ was clearly detected in all of these cells (Fig. 4C).

We then hypothesized that p53-binding to the *CDH1* locus is necessary to maintain *CDH1* expression in A549 cells. We tested this by deleting the ‘b’ region by RNA-guided site-specific DNA cleavage^32^ (Supplementary Figure S3B). After selection of cells successfully transfected with the targeting vectors, approximately 50% efficacy of the deletion was observed both in A549 cells and in MCF7 cells, implying that this deletion might have been achieved monoallelically in these cells (Fig. 4D). Nevertheless, this deletion of the p53-binding site significantly abolished E-cadherin expression both at the protein and mRNA levels in A549 cells, but not in MCF7 cells (Fig. 4E,F), to be consistent with the above hypothesis.

### H3K27ac enables p53-binding to the *CDH1* locus

**Figure 5:**
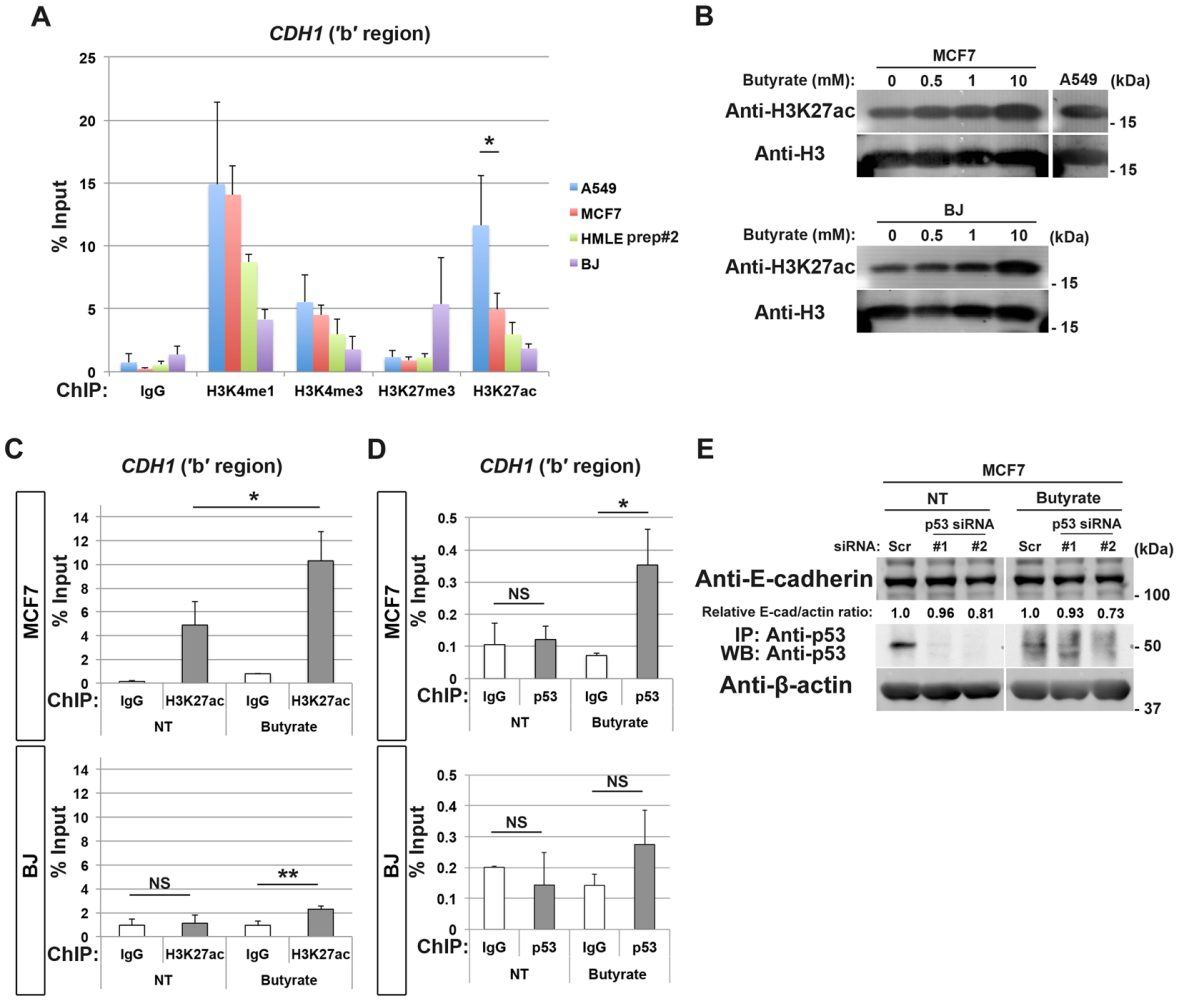
Butyrate promotes p53-binding to the *CDH1* locus in EMT-resistant cells without affecting their integrity. (**A**) A549 cells, MCF7 cells, HMLE prep#2 cells, and BJ cells were subjected to ChIP using antibodies against the indicated modified histones, followed by quantitative RT-PCR analysis with the primers located at the ′b′ region of *CDH1*. Data are means ± SD of 3 independent experiments. *P < 0.05 (Student *t*-test). (**B**) MCF7 cells or BJ cells were treated with the indicated concentrations of butyrate for 24 h. 1.5 µg of chromatin fractions of these cells or non-treated A549 cells were subjected to immunoblot analysis with the indicated antibodies. An uncropped image of this immunoblot of A549 chromatin is also used in Supplementary Figure S4B. (**C**) ChIP was performed using non-treated (NT) or butyrate-treated (10 mM, 48 h) MCF7 cells or BJ cells with a normal rabbit IgG or anti-H3K27ac antibody, followed by quantitative RT-PCR analysis with primers located at the ′b′ region of *CDH1*. Data are means ± SD from 3 independent experiments. **P < 0.01, *P < 0.05; NS, not significant (Student *t*-test). (**D**) ChIP was performed using non-treated (NT) or butyrate-treated (10 mM, 48 h) MCF7 cells or BJ cells with a normal rabbit IgG or anti-p53 antibody, followed by quantitative RT-PCR analysis with primers located at the ′b′ region of *CDH1*. Data are means ± SD from 3 independent experiments. *P < 0.05, NS, not significant (Student *t*-test). (**E**) MCF7 cells transfected with scramble (Scr) or p53 (#1 or #2) siRNA were then treated with or without (NT) 10 mM butyrate for 24 h. Whole cell extracts were then subjected either to immunoblot analysis with antibodies to E-cadherin and β-actin, or to immunoprecipitation with antibodies to p53 (mouse monoclonal) before being subjected to immunoblot analysis with antibodies to p53 (rabbit monoclonal). Quantitative western blotting of E-cadherin and β-actin (E-cad and actin, respectively) was performed with the infrared fluorescence imaging system on an Odyssey imager, and normalized E-cad/actin ratios are indicated. Western blots were cropped for clarity; uncropped images are shown in Supplementary Figure S5.

We next sought to understand a possible mechanism that determines whether or not p53 binds to the *CDH1* nucleotide region. In the earlier experiments, we analysed epigenetic statuses of the *CDH1* TSS region. However, this TSS region is not overlapped with the ‘b’ region of the *CDH1* locus (see Fig. 4A or Supplementary Figure S3A). We therefore analysed epigenetic statuses of the ‘b’ region by a ChIP-qPCR method; and found that this region exhibits high-H3K27ac in A549 cells, but does much lower H3K27ac levels in MCF7 cells, HMLE prep#2 cells, and BJ cells (Fig. 5A). High-H3K27ac, together with high-H3K4me1, is a hallmark of a strong enhancer^15^. A549 cells also showed high-H3K4me1 levels at this region (Fig. 5A). Therefore, the ‘b’ region appeared to possess a strong enhancer property in A549 cells, which is thought to be coupled with an open chromatin configuration.

We then sought to understand whether high levels of H3K27ac at the ‘b’ region of the *CDH1* locus are causative to allow p53-binding to this locus. Sodium butyrate may increase cellular acetyl-CoA levels and thereby promote histone acetylation^33^. We found that MCF7 cells and BJ cells both show an overall increase in H3K27ac levels in response to butyrate, as detected by immunoblotting of histones with an anti-H3K27ac antibody (Fig. 5B). However, only MCF7 cells, but not BJ cells, showed a significant increase in H3K27ac at the ‘b’ region (Fig. 5C), to a level almost comparable to those observed in A549 cells (Fig. 5A). Butyrate may downregulate cellular levels of p53^34^, which we also observed both in MCF7 cells and BJ cells (Supplementary Figure S4A). Nevertheless, a clear binding of endogenous p53 to the ‘b’ region was observed in MCF7 cells, but not in BJ cells, upon butyrate treatment (Fig. 5D). Therefore, H3K27ac levels appeared to be a hallmark that determines the bindability of p53 to the *CDH1* locus. These results simultaneously indicated that the *CDH1* ‘b’ region may exhibit plasticity even in EMT-resistant cells, as in EMT-prone cells. In contrast, histone modifications of this epithelial-specific region were not notably affected by butyrate in *bona fide* fibroblasts, implying that these ‘professional’ mesenchymal cells might have a superior mechanism to tightly close the epithelial gene loci like *CDH1*.

It was reported that hydroxycitrate (HC), which acts as an inhibitor of ATP citrate lyase, as well as a low pH of the culture medium, reduces histone acetylation of HeLa cells^35^. However, we were unable to reduce histone acetylation of A549 cells by HC or by low pH culture conditions (Supplementary Figure S4B and C); and were hence unable to test directly whether the reduced H3K27ac at the *CDH1* locus blocks p53-binding.

### Butyrate does not dampen the epithelial integrity of EMT-resistant cells

The above findings then led us to explore whether the butyrate-induced p53-binding can convert MCF7 cells to be dependent on p53 to maintain *CDH1* expression. For this, we silenced *TP53* in MCF7 cells and cultured them in the presence of butyrate; and found that their expression of E-cadherin was still strictly maintained (Fig. 5E). Therefore, the property of MCF7 cells, in being independent of p53 for their *CDH1* expression, did not seem to be affected by butyrate, even if high-H3K27ac and p53-binding are induced at the *CDH1* locus.

## Discussion

**Figure 6:**
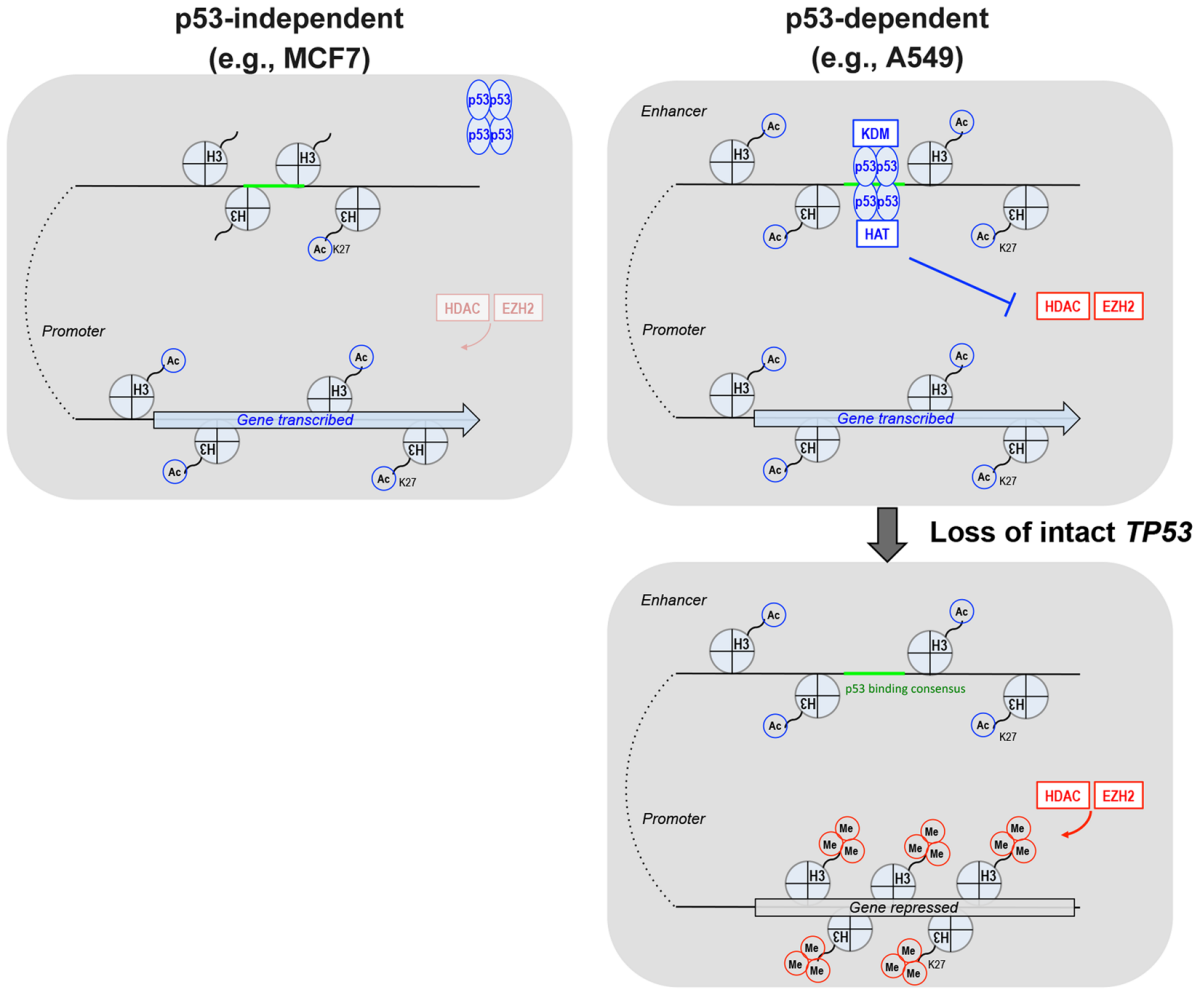
Illustration of the two basic types of epithelial cells in terms of p53-dependency. Specific metabolic states and/or expression of oncogenes may cause the transition of epithelial cells from being p53-independent to p53-dependent, in which p53 maintains epithelial gene expression through the actions of H3K27me3-specific histone lysine demethylase(s) (KDM) and histone acetyltransferase(s) (HAT)^40^.

In this study, we found that histone modifications around the *CDH1* locus, especially at the TSS and the specific p53-binding site, differ significantly among different epithelial cells, as seen between the EMT-prone A549 cells and the EMT-resistant MCF7 cells; and that such differences in histone modifications are linked to the necessity of p53-binding to the *CDH1* locus to maintain its expression (see Fig. 6). We moreover demonstrated that different preparations of HMLEs exhibit different requirement for p53 in their E-cadherin expression (Fig. 1), although epigenetic fluctuations of prep#1 cells have prevented us from their further epigenetic analysis. Therefore, it is likely that the p53-dependent and independent epithelial integrity may co-exist even within the same tissues, although this notion should be confirmed with normal cell populations, without being immortalized in culture or being transformed. Whether the p53-binding-dependent epithelial integrity as seen in A549 cells operates in normal cell populations also remains to be investigated.

Our results indicate that the promoter region of the *CDH1* locus of EMT-prone cells needs p53 to continuously antagonize EZH2-mediated H3K27me3 deposition, in spite of its intrinsic strong enhancer/active promoter property. In contrast, the same *CDH1* region of EMT-resistant epithelial cells does not show the typical strong enhancer/active promoter property, and does not need p53, nor needs to antagonize histone methylation to maintain *CDH1* expression. In the process to antagonize EZH2, p53 seems to utilize H3K27me3-specific demethylase(s). Although p53 has been shown to interact with JMJD3/KDM6B demethylase^22^, we still do not know what demethylase(s) are involved in this process. Likewise, we have yet to fully describe biological significance, as well as mechanisms, of the dynamic antagonism between EZH2 and p53 at the *CDH1* locus in EMT-prone cells (also see later). On the other hand, it is possible that an intrinsically high activity of *CDH1* transcription in EMT-resistant MCF7 cells may be able to outcompete PRC2 binding to this locus^36^.

The *CDH1* locus in EMT-resistant cells was sensitive to butyrate, resulting in high-H3K27ac, whereas this locus in *bone fide* fibroblasts was insensitive to butyrate. Nevertheless, butyrate did not compromise the epithelial integrity of EMT-resistant cells, even if they lose p53 in the presence of butyrate. Butyrate can increase cellular acetyl-CoA levels, and cellular acetyl-CoA levels affect nuclear histone acetylation, as earlier mentioned. Therefore, such a property of EMT-resistant cells seems to be important to resist the easy onset of EMT program, even if these cells lose normal-p53 under metabolic conditions with enhanced acetyl-CoA production. Again, we still do not know the molecular mechanisms therein involved.

One can envisage that p53-dependency or p53-independency for epithelial integrity may relate to whether cells can undergo EMT or not. However, EMT can be induced even in MCF7 cells^37^, although we have tentatively categorized them as EMT-resistant cells in this study. Then, several questions emerge: 1) why some epithelial cells need p53 to constantly protect their epithelial-specific gene loci from being highly H3K27-trimethylated by EZH2, whereas other epithelial cells do not seem to have this mechanism, and, 2) what factor(s) initially determines the dependency and independency of the *CDH1* locus on p53 and its binding to this locus. We are also yet to understand whether the epithelial integrity using the p53-binding and that using the p53-miRNA axis are mutually exclusive with each other, or not. The onset of mesenchymal programs of cancer cells induces not only the high invasive and metastatic activities, but also promotes their resistance to chemotherapies^19^. Understanding the above issues will provide us with further information on the uncharted complexity of histone modifications and their biological meaning with regard to epithelial integrity as well as cancer malignancy development. Such variations of epithelial integrity, which seem to exist even within the same type of tissue, should be taken into account when epithelial tissues are generated from pluripotent stem cells.

## Materials and Methods

### Cell culture

A549 cells, MCF7 cells, H1299 cells, and human diploid fibroblasts (BJ cells) were obtained from authentic stocks (American Type Culture Collection) and cultured under 5% CO_2_ at 37 °C in Dulbecco’s modified Eagle’s medium (DMEM) supplemented with 10% fetal bovine serum (FBS). HMLE cells were generated by introducing SV40 large T antigen and human telomerase reverse transcriptase (*hTERT*) in primary cultures of normal mammary epithelial cells (HMECs) (Lonza). HMLE cells were cultured in Mammary Epithelial Cell Growth Medium (MEGM) (Lonza). No mycoplasma was detected in cultures by 4′,6-diamidino-2-phenylindole (DAPI) staining. Polyclonal cell lines capable of inducible expression of normal-p53 (H1299-TetOn-p53WT cells) were generated by infection of H1299 cells with the corresponding retrovirus together with the rtTA retrovirus (Clontech), followed by selection with puromycin (1 µg/ml) and geneticin (G418, 500 µg/ml).

### Antibodies and reagents

Primary antibodies for immunofluorescence, immunoprecipitation, or immunoblot analysis included the following: rabbit polyclonal anti-vimentin (Abcam); mouse monoclonal anti-E-cadherin, N-cadherin and anti-EZH2 (BD Biosciences); mouse monoclonal anti-γ-tubulin (Sigma-Aldrich); mouse monoclonal anti-JMJD3/KDM6B (Active Motif); rabbit monoclonal anti-ZEB1 (#3396), anti-SNAI1 (#3879), anti-E-cadherin (#3195), anti-p53 (#2527), anti-trimethyl histone H3 (Lys27) (#9733), and anti-acetyl histone H3 (Lys27) (#8173) (Cell Signaling Technology); mouse monoclonal anti-SNAI1 (#3895), anti-p53 (#2524), anti-histone H3 (#14269), and anti-β-actin (#4970) (Cell Signaling Technology). The antibodies used for ChIP assays included the following: normal rabbit IgG (#2729); rabbit monoclonal anti-monomethyl histone H3 (Lys4) (#5326); rabbit monoclonal anti-trimethyl histone H3 (Lys4) (#9751); rabbit monoclonal anti-trimethyl histone H3 (Lys27) (#9733); rabbit monoclonal anti-acetyl histone H3 (Lys27) (#8173); and rabbit monoclonal anti-p53 (#2527) (all from Cell Signaling Technology). CBHA (Merck Millipore) or SAHA (Sigma-Aldrich) was used to inhibit histone deacetylase activities. GSK-J4 (Santa Cruz) was used to inhibit H3K27me3-specific histone demethylase activities. Sodium butyrate (Sigma-Aldrich) was used to increase histone acetylation. Hydroxycitrate (Sigma-Aldrich) was used to reduce histone acetylation.

### Plasmid construction and lentiviral gene transduction

The cDNA for wild-type human p53 (p53WT) was cloned into pRetroX-Tight-Pur (Clontech). Retroviruses with the vesicular stomatitis virus–G (VSV-G) envelope were produced by transfection of GP2-293 cells (Clontech) with the pRetroX construct and pVSV-G (Clontech) using the Lipofectamine LTX reagent (Invitrogen). pX335-U6-Chimeric_BB-CBh-hSpCas9n(D10A) was a gift from Feng Zhang (Addgene plasmid #42335). pINTO-NFH::mEzh2 was a gift from Roberto Bonasio (Addgene plasmid #65925). Lentiviruses expressing shRNA targeting *EZH2* (Mission shRNA, cat. #TRCN0000010475, Sigma-Aldrich) were produced by transfection of 293FT cells (Invitrogen) with the shRNA constructs on the pLKO.1-puro vector, the envelope plasmid pMD2.G (Addgene #12259), and the packaging plasmid psPAX2 (Addgene #12260), using Lipofectamine LTX (Invitrogen) according to the manufacturer’s instructions.

### RNA interference (RNAi)

For the transfection of siRNA targeting human *TP53*, *EZH2* or *JMJD3/KDM6B*, cells were plated at 15%-20% confluence in 6-well plates, cultured overnight, and then incubated in the presence of 150 pmol of siRNA duplexes and 4 µL of RNAi MAX (Invitrogen). They were then cultured in complete medium for 24 h before a second round of siRNA transfection. For the reverse transfection of siRNA, a mixture of 150 pmol of EZH2 siRNA and 4 µL of RNAi MAX (Invitrogen) in Opti-MEM (Invitrogen) and A549 cells (6 × 10^4^) in complete culture medium was added to each well of a 6-well plate. Alternatively, cells were infected with lentiviruses expressing shRNA targeting *EZH2*. The cells were then transfected with scramble or p53 siRNA on two consecutive days. Duplex oligonucleotides targeting *TP53* mRNA (5′-CCGCGCCAUGGCCAUCUACdTdT-3′: p53 #1, and 5′-GACUCCAGUGGUAAUCUACdTdT-3′: p53 #2) were chemically synthesized and purified by Japan BioService. shRNA targeting a coding sequence (5′-GACUCCAGUGGUAAUCUACU-3′: p53 #3, Addgene #10672) or the 3′-untranslated region (5′-GAGGGAUGUUUGGGAGAUGUA-3′: p53 #4, Mission shRNA, cat. No. TRCN0000342261, Sigma-Aldrich) of *TP53* mRNA was also used. The control siRNA sequences were designed by scrambling the irrelevant siRNA sequences. Stealth siRNAs targeting a coding sequence of human *EZH2* mRNA (5′-GACCACAGUGUUACCAGCAUUUGGA-3′: EZH2 #1), human *JMJD3/KDM6B* mRNA (5′-GGGAAGUUUCGAGAGUCCUACCUUU-3′: JMJD3 #1, and 5′-CGGCACACAGCAGUCGGAAACCGUU-3′: JMJD3 #3), or a stealth RNAi negative control duplex (as a control) were obtained from Invitrogen. Alternatively, lentiviruses expressing shRNA for the 3′-untranslated region of *EZH2* mRNA (5′-GAAACAGCUGCCUUAGCUUCA-3′: EZH2 #2) was used (Mission shRNA, cat. #TRCN0000010475, Sigma-Aldrich).

### Matrigel invasion assay

A549 cells (4.0 × 10^4^) or MCF7 cells (2.0 × 10^5^) transfected with a control or p53 siRNA sequences were deprived of serum for 15 h before being transferred to a BioCoat Matrigel Invasion Chamber (BD Biosciences), and then cultured for 24 h. The cells were then fixed with 3.7% formaldehyde in phosphate buffered saline (PBS) for 30 min and washed with PBS, and those that had invaded the Matrigel were stained with crystal violet. After washing the cells with PBS at least five times, the number of those in three different regions on the lower surface of the filter was quantified.

### Immunofluorescence analysis

For immunofluorescence analysis, cells cultured on coverslips were fixed with 4% paraformaldehyde in PBS, permeabilized with 0.1% Triton X-100 in PBS for 10 min, and incubated with primary antibodies for 60 min at room temperature. They were then washed with PBS and incubated with Alexa Fluor 488-conjugated secondary antibodies (Molecular Probes) for 30 min. The cells were also stained with Texas Red–phalloidin (Molecular Probes) to detect F-actin and with DAPI to visualize nuclei. The cells were finally washed with PBS, mounted on glass slides, and examined with a confocal laser-scanning microscope using a CFI Plan Apo VC 60 × H oil-immersion objective with an NA of 1.4, and analysed with the attached software (Model A1R with NIS-Elements, Nikon).

### Live-cell imaging analysis

Live-cell imaging was performed with a confocal laser-scanning microscope using a CFI Plan Apo VC 40 × H oil-immersion objective with an NA of 1.0 under 5% CO_2_ at 37 °C. Cells were exposed to Hoechst 33342 at 1 µg/ml for 30 min before observation to visualize nuclei, and their trajectories were analysed with the attached software (Model A1R with NIS-Elements, Nikon).

### Reverse transcription (RT)-PCR analysis

Total RNA was extracted from cells using Trizol reagent (Invitrogen), purified with Direct-zol RNA Miniprep kit (Zymo Research), and aliquots (0.5 to 1 μg) of the RNA were subjected to RT with SuperScript II or SuperScript IV polymerase (Invitrogen). TaqMan RT-PCR primers for *CDH1*, *SNAI1*, *ZEB1*, and *ACTB* were obtained from Applied Biosystems for quantitative PCR analysis. Quantitative PCR analysis was performed using the 7300 Fast Real-Time PCR System (Applied Biosystems), and the amount of target mRNA was normalized by that of *ACTB* mRNA.

### Chromatin immunoprecipitation (ChIP)

A549 cells, H1299 cells, MCF7 cells, HMLE cells, and BJ cells were subjected to ChIP using the Simple ChIP Enzymatic Chromatin IP Kit (Cell Signaling Technology) according to the manufacturer’s instructions. Immunoprecipitated DNA was evaluated either by quantitative or semi-quantitative PCR.

### Primers used for the ChIP assay

Sequences of the PCR primers used for the ChIP assay in Fig. 2, 4, or 5 are the following: 5′-AGTGCTATCAACATTCCCATTTTACA-3′ as the forward, and 5′-GGCTGAGGTAGGAGGATCATTTG-3′ as the reverse, with 6-carboxyfluorescein (FAM)- and tetramethylrhodamine (TAMRA)-labeled 5′-ACTTCACTCCAGCCTGGATTCACTTCTGGC-3′ as the probe to quantify the ′b′ region of *CDH1*; 5′-TGCCAAGAAAGGTCGTAAATAGGA-3′ as the forward, and 5′-CTTAGACCGGGAATGCACCAC-3′ as the reverse, with FAM- and TAMRA-labeled 5′-CGCTCCCACCTCCTCCGACCTCAC-3′ as the probe to quantify the TSS region of *CDH1*; 5′-AGCAGGCTGTGGCTCTGATT-3′ as the forward, and 5′-CAAAATAGCCACCAGCCTCTTCT-3′ as the reverse, with FAM- and TAMRA-labeled 5′-GCCGTCAGGAACATGTCCCAACATGTTGAG-3′ as the probe to quantify the 5′ region of *CDKN1A*. The sequences of the PCR primers used in Figure S3A to semi-quantify the *CDH1* locus were the following: 5′-CGGCCCTTCACCTAATTCTT-3′ as the forward, and 5′-GCCTTTGAGTCAGAGCCACA-3′ as the reverse for region “a”; 5′-GAGGAAACCAAGATGGTGGA-3′ as the forward, and 5′-AGCACTGGAGCCACTTCACT-3′ as the reverse for region “b”; 5′-TTGGAGGGGACAGGTAGATG-3′ as the forward, and 5′-CTCCACCCCAATGCATAGTT-3′ as the reverse for region “c”; 5′-GCAGCACTCAAAGCACTCAC-3′ as the forward, and 5′-AATACCCCCACCCTCAACTC-3′ as the reverse for region “d”; 5′-GGTTGCATGGGGAGATAATG-3′ as the forward, and 5′-CTTTGGGGCACCTCATCTTA-3′ as the reverse for region “e”.

### ChIP Sequencing

Template preparation for next generation sequencing was performed using the immunoprecipitated DNA and TruSeq ChIP Seq kit (Illumina). Sequencing was performed on the HiSeq. 2500 platform by 36-base-single-end sequencing. We used Model-based Analysis for ChIP-Seq (MACS) with input DNA as a control, to identify the following two types of regions of enrichment: (1) narrow peaks of contiguous enrichment (narrowPeaks) that pass a Poisson *p*-value threshold of 0.00001 (MACS v1.4.2, default parameters), and (2) broader regions of enrichment (broadPeaks) that pass a *q*-value threshold of 0.05 (broad peak mode of MACS v2.1.0). For the broad-range histone marker, H3K27me3, we used the broadPeak representation of MACS v2.1.0 peak caller^38^.

### Genome editing

We designed four different guide RNAs (gRNAs) targeting a 20-bp site (protospacer) in the human *CDH1* locus that precedes an NGG trinucleotides, which is the requisite protospacer-adjacent motif (PAM) (Supplementary Figure S3B). Each sequence was then cloned into the pX335-U6-Chimeric_BB-CBh-hSpCas9n(D10A) vector which coexpresses a gRNA and a nickase. A homology repair template was designed to delete the p53-binding consensus motif and insert a Piggybac transposon cassette encoding a selection marker (puromycin N-acetyltransferase gene) and a fluorescent reporter (Ruby3 fused to a PEST sequence) into a TTAA sequence nearby. These constructs were transfected into the cells using Lipofectamine LTX (Invitrogen) according to the manufacturer’s instructions. The cells with successful genome editing were selected by culturing them in the presence of 0.2 µg/mL of puromycin. The cassette was removed by transposase-mediated excision; cells were transfected using pExPBase, which encodes an integration-defective, excision-competent Piggybac transposase mutant^39^, to restore the original TTAA sequence. Each genomic status was confirmed by PCR (Supplementary Figure S3B) or by sequencing (Fig. 4D).

### Chromatin isolation

Chromatin fractions were prepared by resuspending the cells in lysis buffer (10 mM 4-(2-hydroxyethyl)-1-piperazineethanesulfonic acid (HEPES) pH 7.4, 10 mM KCl, 0.05% NP-40) supplemented with a protease inhibitor cocktail (Roche) and 5 mM sodium butyrate, and incubated on ice for 20 min. The lysates were then centrifuged at 14,000 rpm for 10 min at 4 °C. The supernatant (cytosolic fraction) was removed and the pellet (nuclei) was resuspended in 0.2 N HCl for acid-extraction and incubated on ice for 20 min. The lysates were then centrifuged at 14,000 rpm for 10 min at 4 °C. The supernatants (acid-extracted proteins) were neutralized with 1.5 M Tris-HCl (pH 8.8).

### TCGA analysis

RNA sequencing data sets were obtained from TCGA. According to p53 mutational status, we grouped tumor samples into (1) wild-type (tumors without a detectable p53 mutation) or (2) mutants (tumors with p53 missense mutations, nonsense mutations, insertions/deletions, or truncations). Original RNA expression values from TCGA data sets available as of April 2015 (breast carcinoma) or July 2015 (lung adenocarcinoma) were converted to log2 values before analysis. The Spearman rank correlation test was used to calculate *P*-values.

### Statistical analysis

Quantitative data are presented as the mean ± SD. Comparisons between groups were performed using a two-tailed Student *t*-test with equal variance. *F*-test was conducted before Student *t*-test to compare the variance of two samples. A *P*-value of less than 0.05 was considered to indicate a statistically significant difference between two groups.

### Data Availability

The datasets generated during and/or analysed during the current study are available from the corresponding author on reasonable request.

## Electronic supplementary material

Supplementary Information
