# Supplementary material for: Necessity of p53-binding to the CDH1 locus for its expression defines two epithelial cell types differing in their integrity
Source: Sci Rep. 2018 Jan 25;8:1595. doi: 10.1038/s41598-018-20043-7 (PMC5785525; doi:10.1038/s41598-018-20043-7)

Supplementary Information

Necessity of p53-binding to the *CDH1* locus for its expression defines two epithelial cell types differing in their integrity

Tsukasa Oikawa,[1] Yutaro Otsuka,[1] Yasuhito Onodera,[1] Mei Horikawa,[1] Haruka Handa,[1] Shigeru Hashimoto,[1] Yutaka Suzuki,[2] and Hisataka Sabe[1]*

[1]Department of Molecular Biology, Graduate School of Medicine, Hokkaido University, North 15, West 7, Kita-ku, Sapporo, Hokkaido 060-8638, Japan

[2]Laboratory of Functional Genomics, Department of Medical Genome Sciences, Graduate School of Frontier Science, The University of Tokyo, 5-1-5 Kashiwanoha, Kashiwa, Chiba 277-8561, Japan

*To whom correspondence should be addressed

Tel.: +81-11-706-5044 (ext. 5044); Fax: +81-11-706-7865

E-mail: sabeh@med.hokudai.ac.jp

Supplementary figure legends

Figure S1 (related to Figure 2). A549 cells transfected with scramble (Scr) or JMJD3/KDM6B (JMJD3#1 or JMJD3#3) siRNA were subjected to immunoblot analysis with the indicated antibodies.

Figure S2 (related to Figure 3). A, A549 cells were subjected to ChIP-Seq using antibodies against the indicated modified histones, after the transfection of scramble (Scr RNAi) or p53 (p53 RNAi) siRNA. IGV genome browser screenshots of MACS-called peaks are shown. B, Dot plots of TCGA RNA expression profiles of *EZH2* in lung cancer or breast cancer with wild-type (wt) p53 or mutant (mut) p53. Bars represent means ± SD. *P*-values calculated by the Student *t*-test are indicated.

Figure S3 (related to Figure 4). Strategy for deletion of the p53-binding motif at the *CDH1* locus. A, UCSC Genome Browser view of the ENCODE data at the *CDH1* locus. The *CDH1* locus contains the p53 consensus binding motifs (a-e). Among them, the region with a motif to which p53-binding was confirmed (b) is magnified with the sequence: the p53 binding consensus is shown in red; the "TTAA" sequence used for scar-less genome editing in (B) is shown in blue; and the primer sequences used to confirm genome status in (B) are underlined. A549 cells or BJ cells were subjected to ChIP using a normal rabbit IgG or anti-p53 antibody, followed by semi-quantitative RT-PCR analysis with the primers located at the p53 consensus binding motifs (a-e) across the *CDH1* locus. B, Schematic representation of the scar-less genome editing

strategy. A homology repair template was designed to contain a gene cassette with the puromycin N-acetyltransferase gene (Puro^r) and Ruby3-PEST red-fluorescent gene, which was then removed upon the expression of an excision-only piggybac transposase after the selection. Note that the original TTAA sequence was restored. Primers used to amplify the modified region are shown as horizontal arrows.

Figure S4 (related to Figure 5). A, Lysates of MCF7 cells or BJ cells treated with (+) or without (−) 10 mM butyrate for 24 h were subjected to immunoblot analysis with the indicated antibodies. B, A549 cells were treated with the indicated concentrations of hydroxycitrate for 24 h. 1.5 μg of chromatin fractions were subjected to immunoblot analysis with the indicated antibodies. C, A549 cells were cultured in the medium containing the indicated concentrations of bicarbonate for 18 h. Before cell lysis, pH of the culture medium (pHe) was measured. 1.5 μg of chromatin fractions were subjected to immunoblot analysis with the indicated antibodies.

Figure S5. Full-length blots used in the main figures.

Supplementary Figure S1

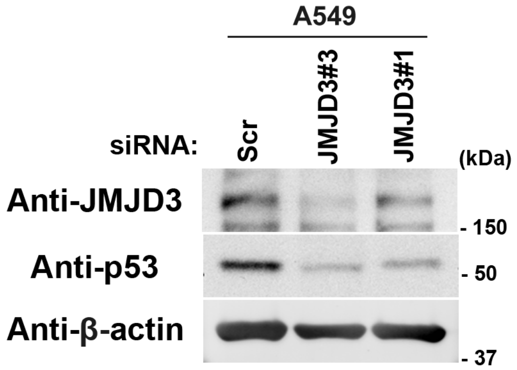

Supplementary Figure S2

A

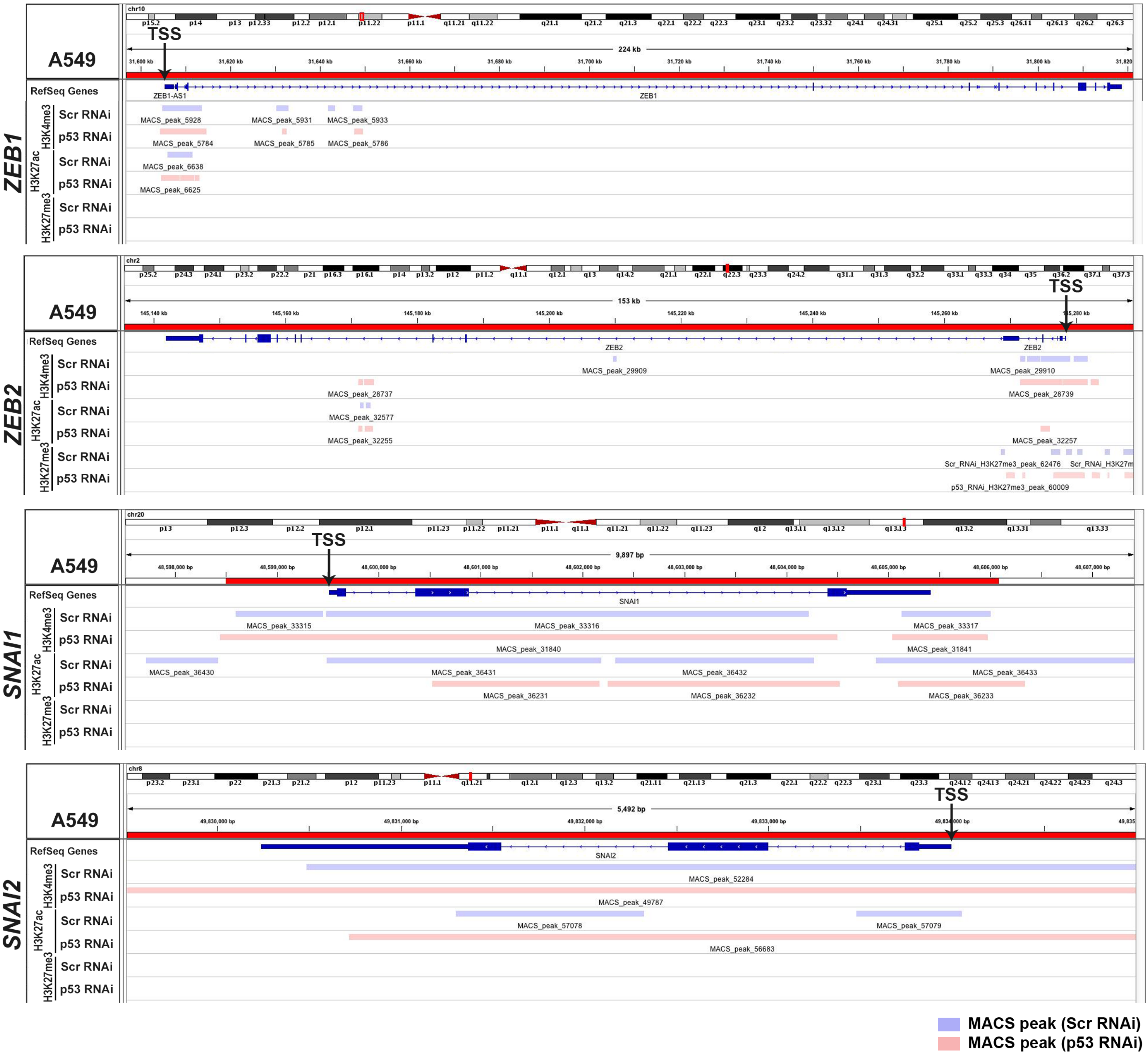

Supplementary Figure S2

A (continued)

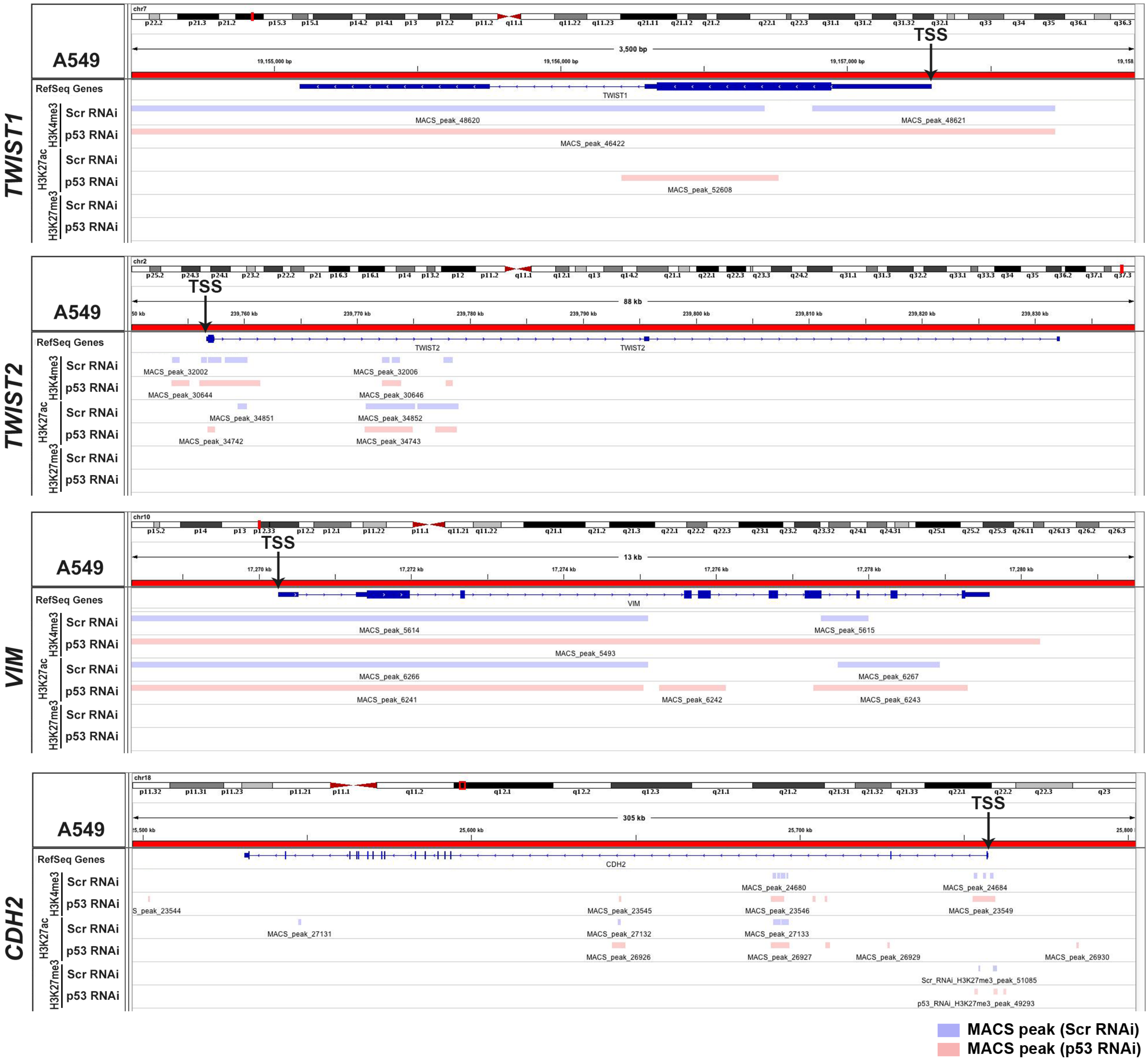

B

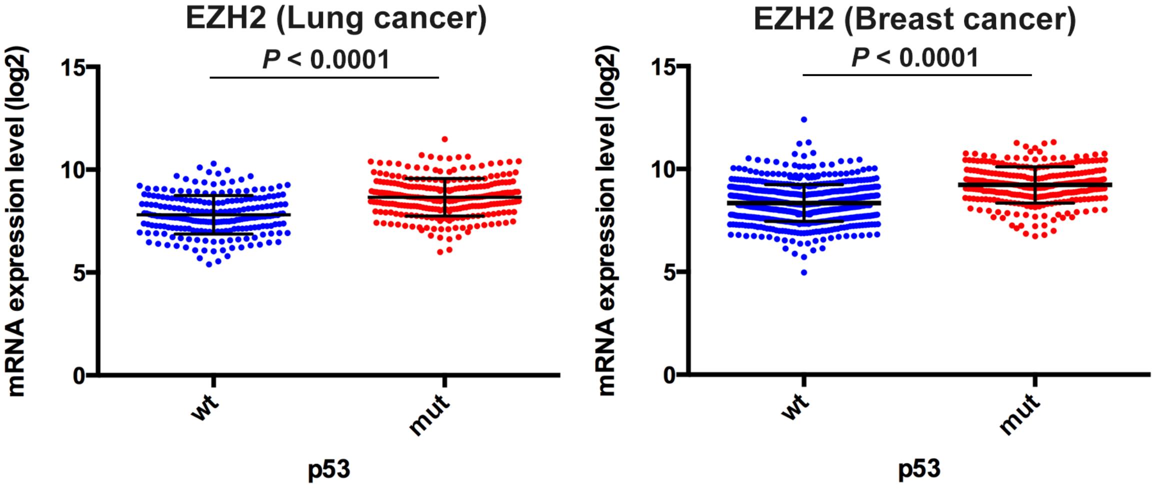

Supplementary Figure S3

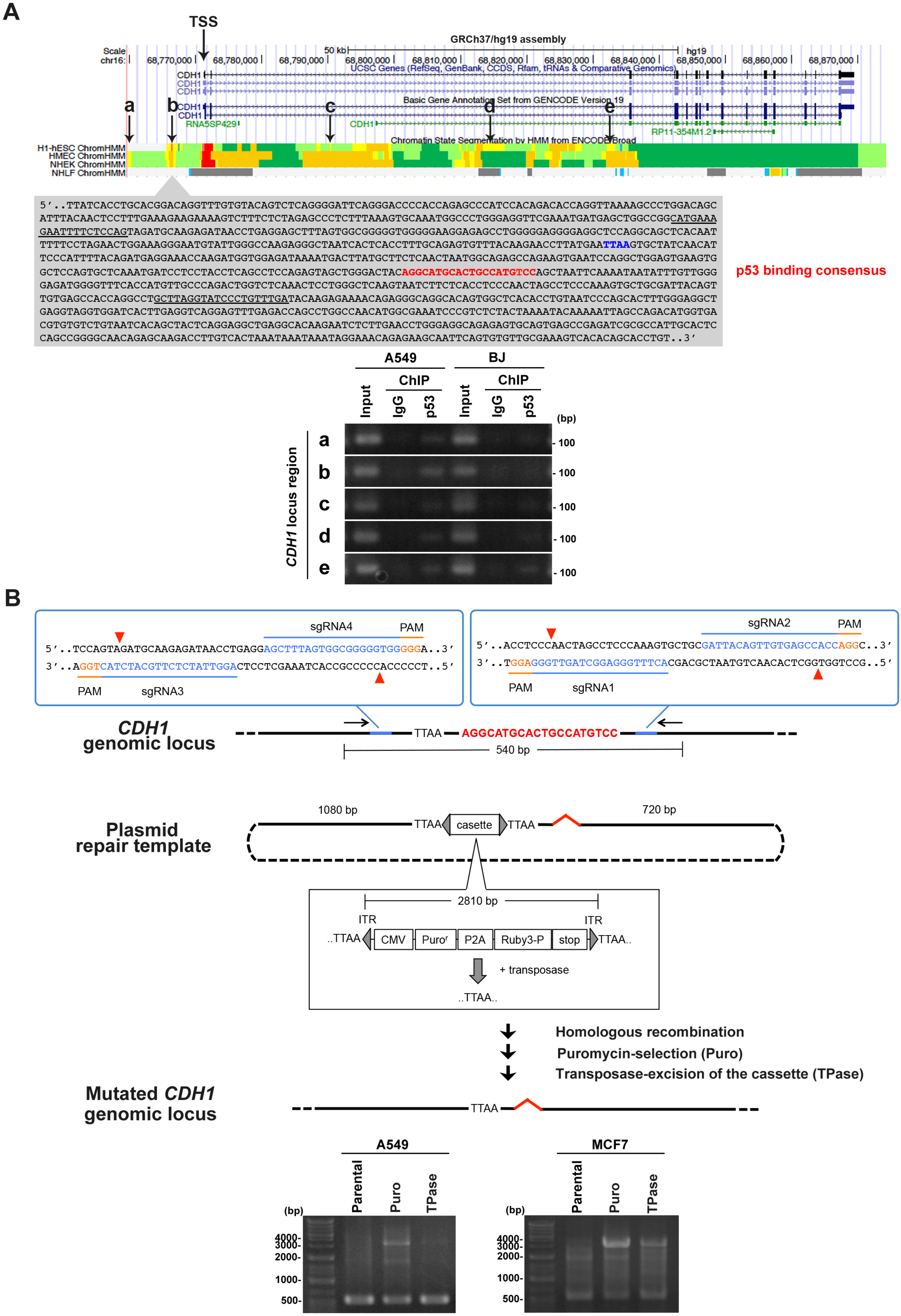

Supplementary Figure S4

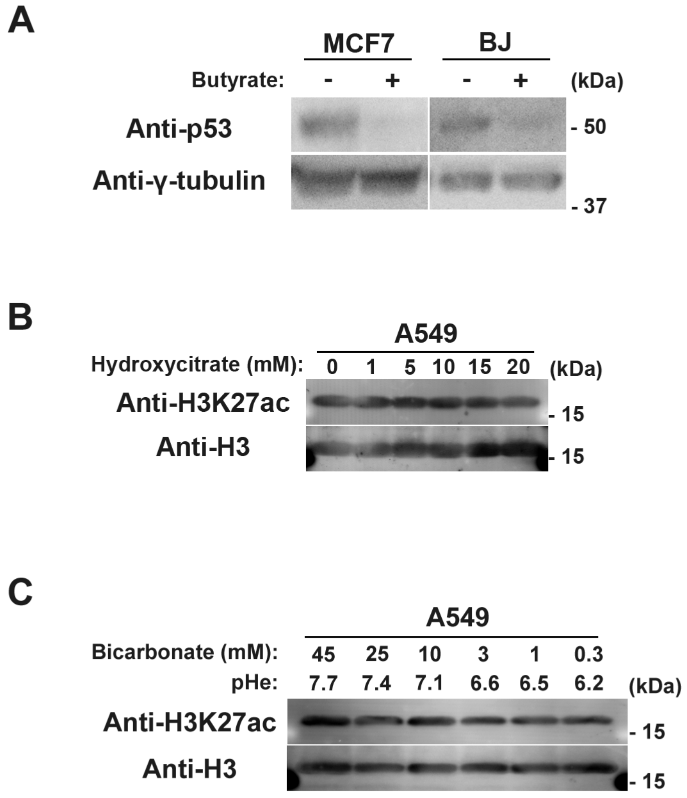

Supplementary Figure S5

Full-length blots used in Figure 1A

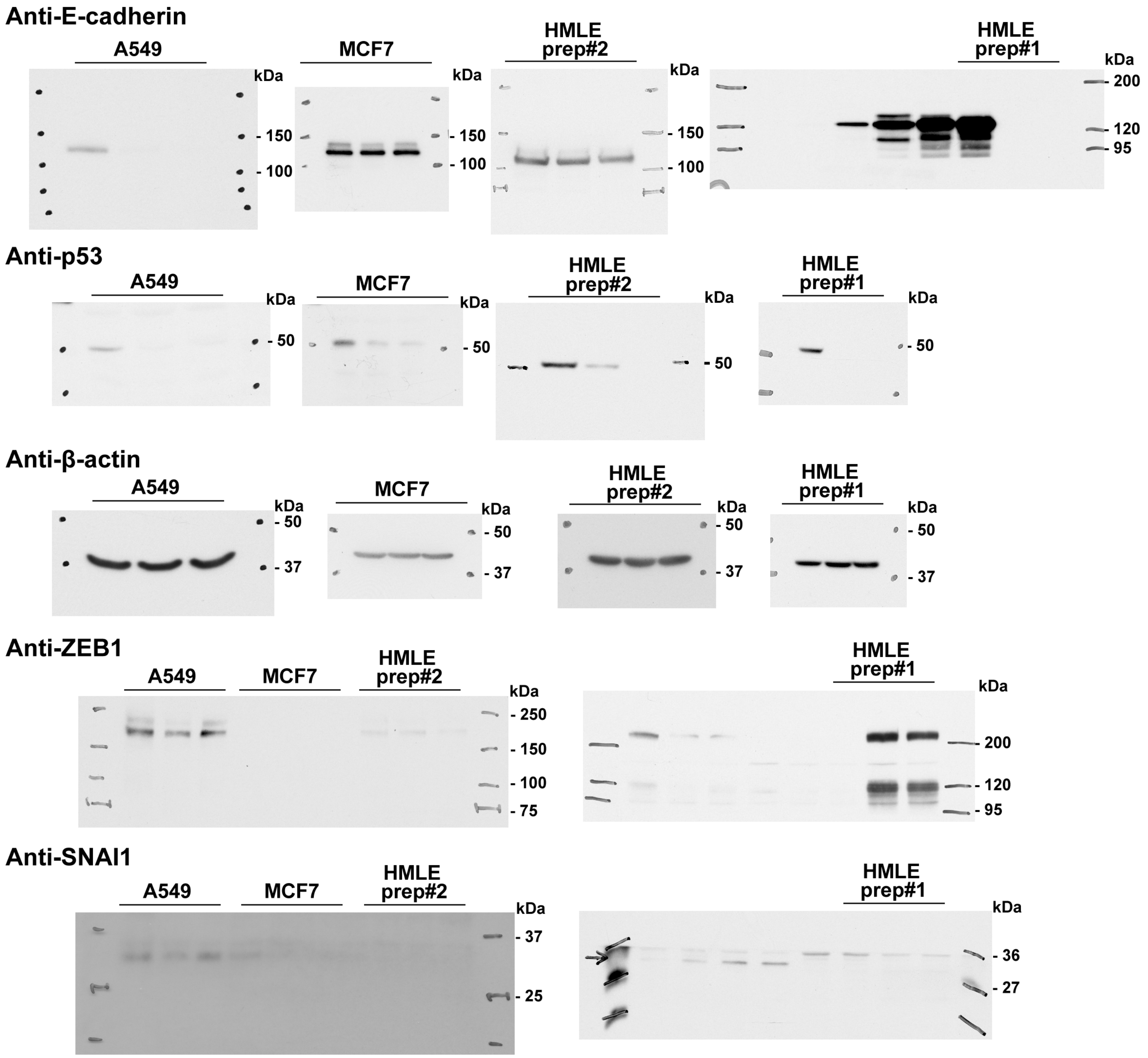

Full-length blots used in Figure 1C

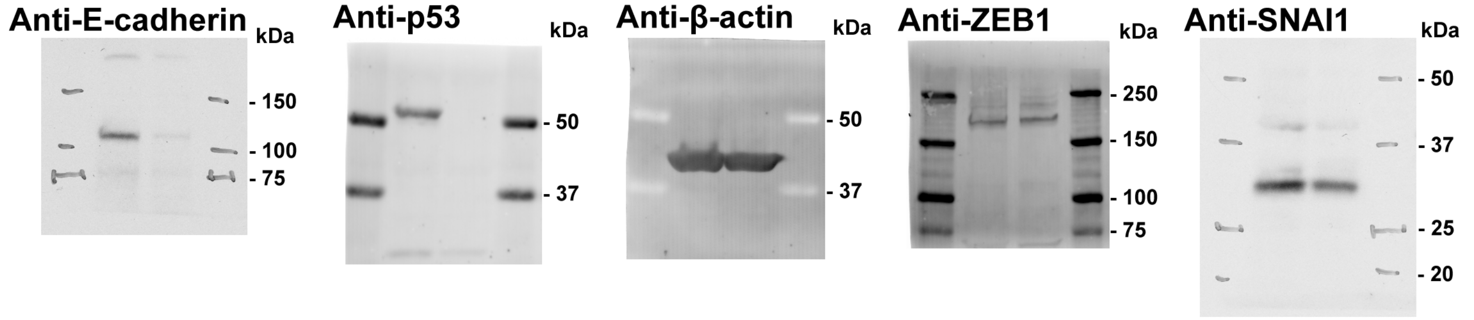

Supplementary Figure S5

Full-length blots used in Figure 2B

Anti-E-cadherin

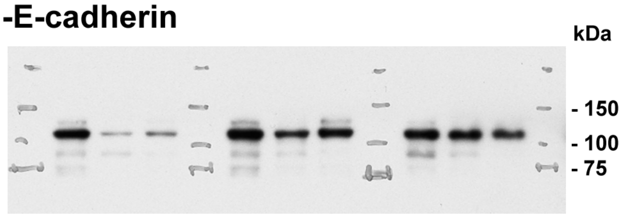

Anti-p53

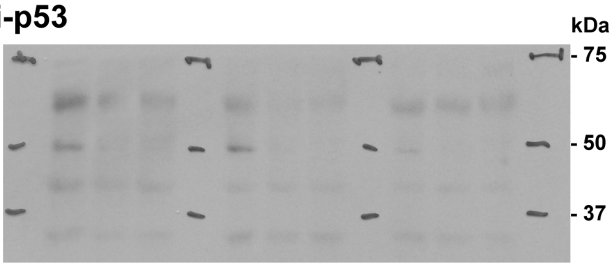

Anti-β-actin

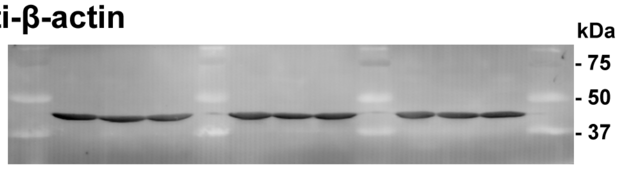

Anti-H3K27ac

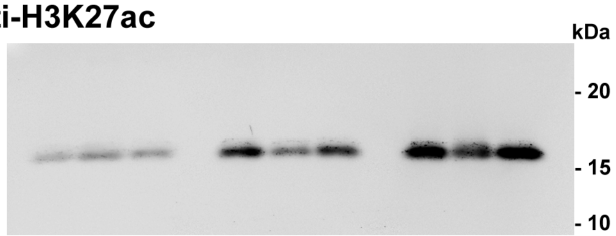

Anti-H3

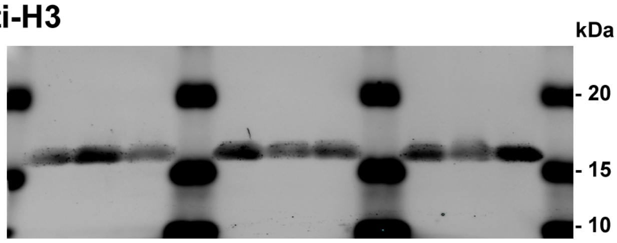

Full-length blots used in Figure 2C

Anti-E-cadherin

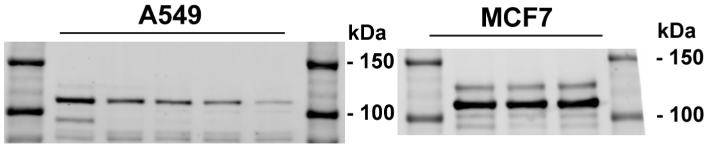

Anti-p53

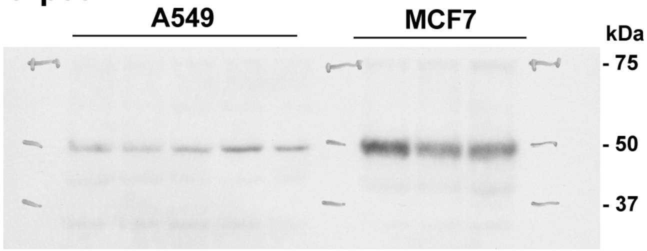

Anti-β-actin

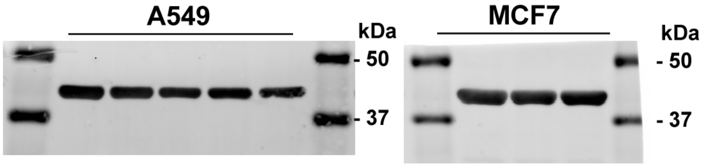

Anti-H3K27me3

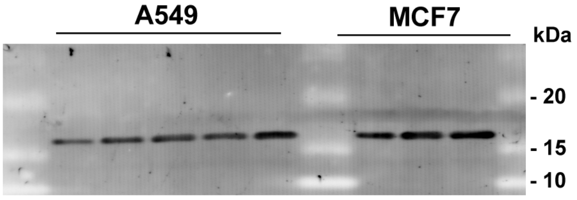

Anti-H3

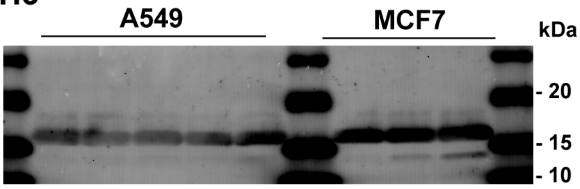

Anti-ZEB1

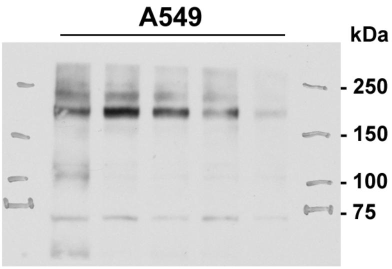

Anti-SNAI1

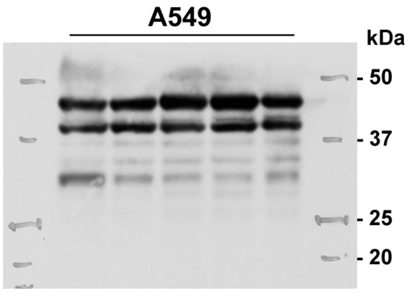

Supplementary Figure S5

Full-length blots used in Figure 3A

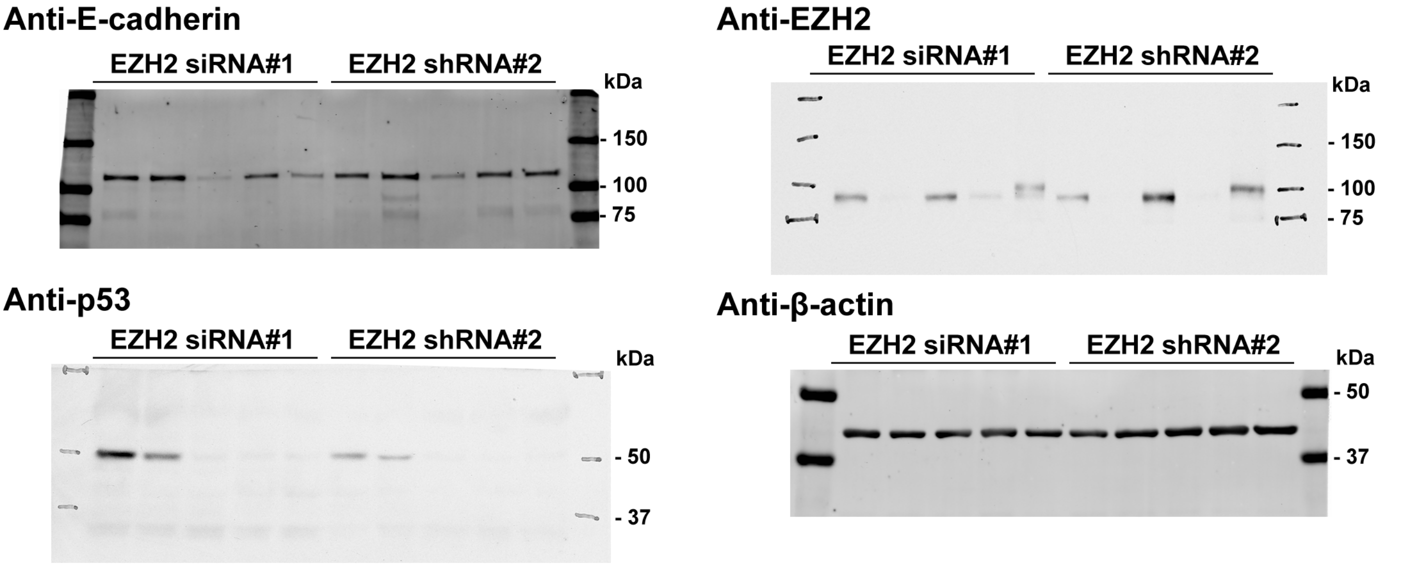

Full-length blots used in Figure 3C

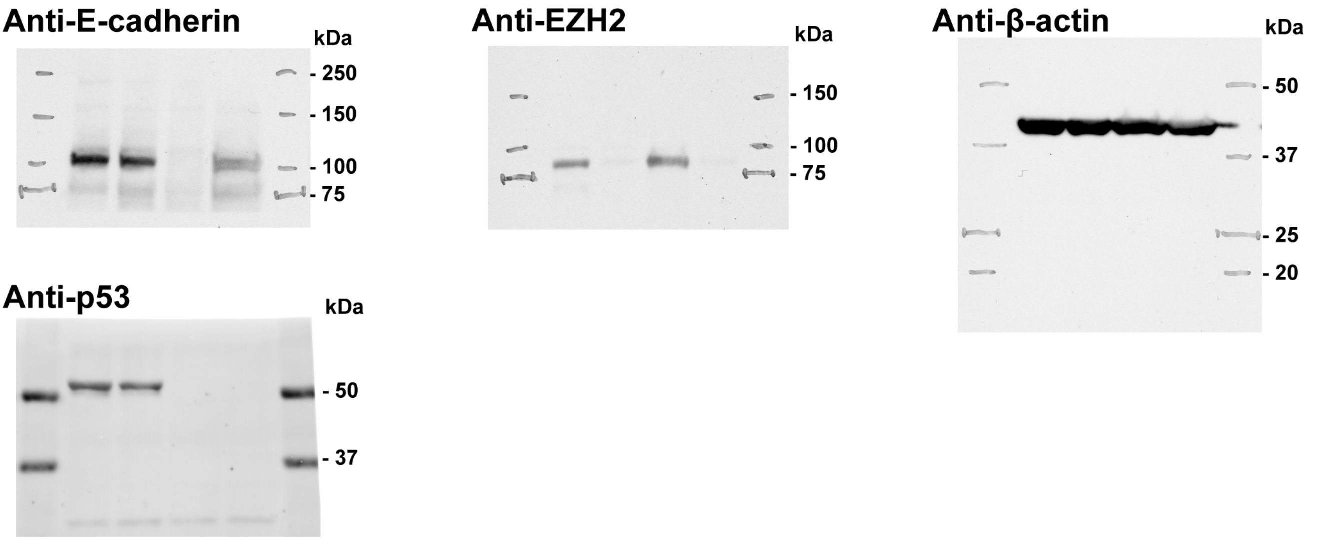

Full-length blots used in Figure 3D

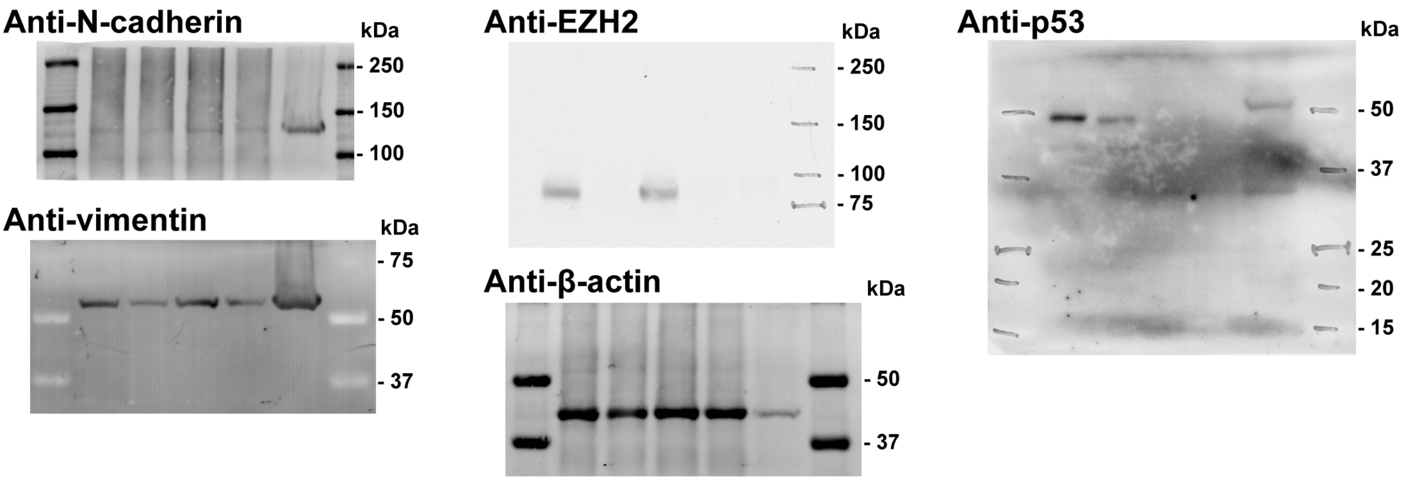

Supplementary Figure S5

Full-length blots used in Figure 4E

Anti-E-cadherin

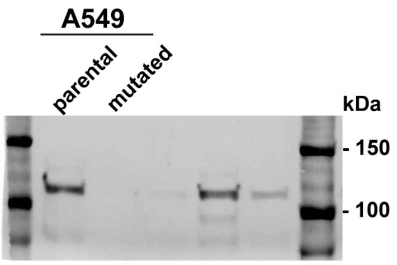

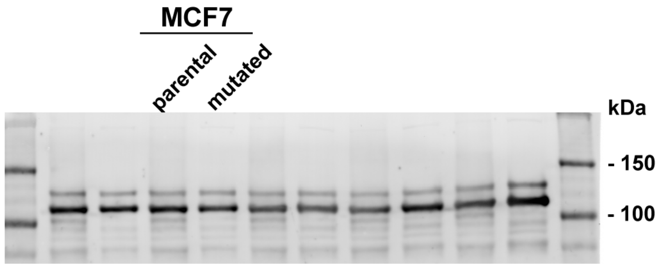

Anti-β-actin

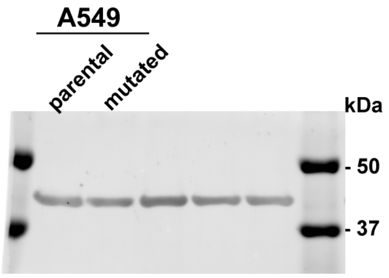

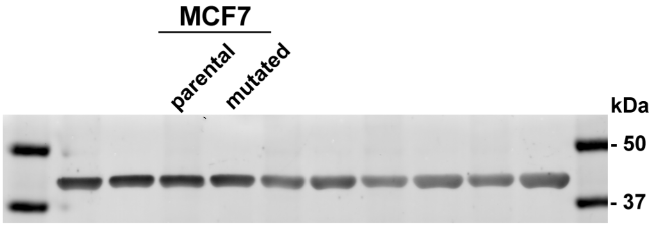

Full-length blots used in Figure 5B

Anti-H3K27ac

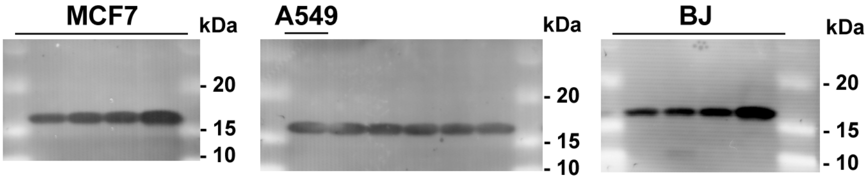

Anti-H3

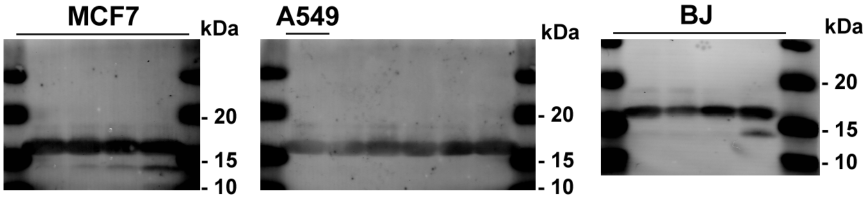

Full-length blots used in Figure 5E

Anti-E-cadherin

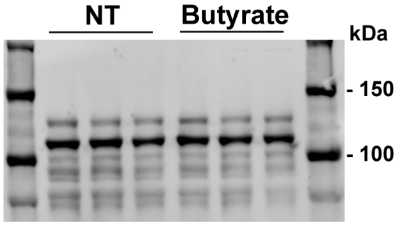

Anti-β-actin

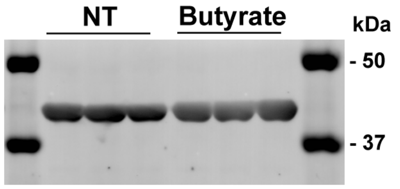

Anti-p53

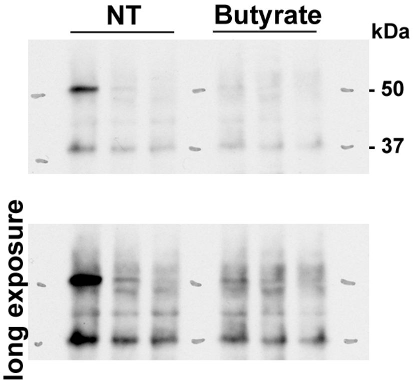

Supplement: Supplementary file 1 — Supplementary Information [file 41598_2018_20043_MOESM1_ESM.pdf]
